# Differential effects of Th1 versus Th2 cytokines in combination with hypoxia on HIFs and angiogenesis in RA

**DOI:** 10.1186/ar3934

**Published:** 2012-08-06

**Authors:** Helene Larsen, Barbara Muz, Tak L Khong, Marc Feldmann, Ewa M Paleolog

**Affiliations:** 1 Nuffield Department of Orthopaedics, Rheumatology and Musculoskeletal Sciences, Kennedy Institute of Rheumatology, University of Oxford, Arthritis Research Campaign Building, 65 Aspenlea Road, London W6 8LH, UK; 2 Department of Radiation Oncology, Cancer Biology Division, St. Louis School of Medicine, Washington University, 4511 Forest Park Avenue, St. Louis, MO 63108, USA; 3 Department of Surgery, Pinderfields Hospital Aberford Road, Wakefield WF1 4DG, UK

## Abstract

**Introduction:**

Hypoxia and T-helper cell 1 (Th1) cytokine-driven inflammation are key features of rheumatoid arthritis (RA) and contribute to disease pathogenesis by promoting angiogenesis. The objective of our study was to characterise the angiogenic gene signature of RA fibroblast-like synoviocytes (FLS) in response to hypoxia, as well as Th1 and T-helper cell 2 (Th2) cytokines, and in particular to dissect out effects of combined hypoxia and cytokines on hypoxia inducible transcription factors (HIFs) and angiogenesis.

**Methods:**

Human angiogenesis PCR arrays were used to screen cDNA from RA FLS exposed to hypoxia (1% oxygen) or dimethyloxalylglycine, which stabilises HIFs. The involvement of HIF isoforms in generating the angiogenic signature of RA FLS stimulated with hypoxia and/or cytokines was investigated using a DNA-binding assay and RNA interference. The angiogenic potential of conditioned media from hypoxia-treated and/or cytokine-treated RA FLS was measured using an *in vitro *endothelial-based assay.

**Results:**

Expression of 12 angiogenic genes was significantly altered in RA FLS exposed to hypoxia, and seven of these were changed by dimethyloxalylglycine, including ephrin A3 (EFNA3), vascular endothelial growth factor (VEGF), adipokines angiopoietin-like (ANGPTL)-4 and leptin. These four proangiogenic genes were dependent on HIF-1 in hypoxia to various degrees: EFNA3 >ANGPTL-4 >VEGF >leptin. The Th1 cytokines TNFα and IL-1β induced HIF-1 but not HIF-2 transcription as well as activity, and this effect was additive with hypoxia. In contrast, Th2 cytokines had no effect on HIFs. IL-1β synergised with hypoxia to upregulate EFNA3 and VEGF in a HIF-1-dependent fashion but, despite strongly inducing HIF-1, TNFα suppressed adipokine expression and had minimal effect on EFNA3. Supernatants from RA FLS subjected to hypoxia and TNFα induced fewer endothelial tubules than those from FLS subjected to TNFα or hypoxia alone, despite high VEGF protein levels. The Th2 cytokine IL-4 strongly induced ANGPTL-4 and angiogenesis by normoxic FLS and synergised with hypoxia to induce further proangiogenic activity.

**Conclusion:**

The present work demonstrates that Th1 cytokines in combination with hypoxia are not sufficient to induce angiogenic activity by RA FLS despite HIF-1 activation and VEGF production. In contrast, Th2 cytokines induce angiogenic activity in normoxia and hypoxia, despite their inability to activate HIFs, highlighting the complex relationships between hypoxia, angiogenesis and inflammation in RA.

## Introduction

The inflammatory and invasive rheumatoid arthritis (RA) synovial tissue is characterised by elevated levels of inflammatory T-helper cell 1 (Th1) cytokines such as IL-1β and TNFα (reviewed in [1]), as well as by lowered oxygen tensions ranging between 2.4 and 4.4% oxygen (18 to 33 mmHg) compared with 8.5 to 13.5% (65 to 103 mmHg) in healthy individuals [2]. Hypoxia in RA is thought to arise as a consequence of thickening of the synovial lining and infiltration by cells, predominantly circulating T cells, B cells and macrophages. This eventually leads to formation of a thick multilayered granulation tissue, termed pannus, which has propensity for invasion at the interface of cartilage and bone, resulting in progressive joint and soft tissue destruction [3,4]. Oxygen delivery becomes progressively compromised with increasing distances between the expanding tissue mass of pannus and existing synovial vasculature, resulting in tissue hypoxia. Inflammation and hypoxia support activation of local blood vessels and ongoing angiogenesis in the synovial membrane, which is an important early step in the pathogenesis of RA [5]. Counterintuitively, despite attempts at restoring homeostasis through the process of angiogenesis, tissue hypoxia prevails due to the immaturity and dysfunctional nature of the newly formed vessels [6].

There is considerable evidence to suggest that angiogenesis and chronic inflammation are co-dependent in inflammatory diseases such as RA (reviewed in [7]). For instance, increased blood vessel formation, and hence an increased surface area of vessels, can maintain the chronic inflammatory state through increased production of cytokines and by allowing inflammatory cells to access the inflamed synovial tissue. Moreover, angiogenesis sustains the supply of nutrients and oxygen to the hyperproliferating inflamed RA tissue. Conversely, inflammatory mediators such as Th1 cytokines and growth factors secreted by the infiltrating inflammatory cells are known to have both direct and indirect angiogenic effects on endothelial cells and resident RA synovial cells, respectively [8,9]. These interdependent mechanisms underlying angiogenesis and inflammation in RA explain why biologics targeting inflammatory cytokines also reduce angiogenesis and why induction of angiogenesis is associated with increased synovial inflammation and pannus formation [6,10]. Likewise, angiogenesis blockade has been shown to reduce inflammation [11].

Major transcription factors that may constitute a link between inflammation, hypoxia and angiogenesis are the heterodimeric hypoxia-inducible factor (HIF)-1 and HIF-2. These are best known as the principal mediators of cellular responses to hypoxia, such as angiogenic responses involving the well-known angiogenic factor vascular endothelial growth factor (VEGF) and a plethora of other angiogenic genes [12]. Both HIF isoforms accumulate in circumstances of oxygen deprivation, which is associated with decreased degradation of the HIF-1α and HIF-2α subunits. This enables the transcription of HIF target genes, characterised by the presence of hypoxia-response elements (HRE). HIF-1α is ubiquitously expressed whereas HIF-2α is expressed in a more limited fashion. It is becoming clear that the two isoforms have overlapping as well as unique roles in hypoxia signalling, which are achieved through differential regulation and specific target gene selection. HIF-1α, HIF-2α and VEGF are all overexpressed in the synovial lining and stromal cells in rheumatoid synovia compared with normal synovia [13]. Moreover, the number of HIF-1α-positive cells has been shown to correlate strongly with the number of blood vessels in RA synovial tissue and with inflammatory endothelial cell infiltration, cell proliferation and the synovitis score [14].

In parallel to the oxygen-dependent pathway, HIF-1α and HIF-2α subunits are also regulated by inflammatory cytokines such as IL-1β and TNFα under normoxic conditions via receptor-mediated signals. In contrast to the specific stabilisation of HIF-α protein occurring under hypoxic conditions, Th1 cytokines appear to act on several regulatory levels and have been reported to stimulate HIF-α mRNA synthesis and stability in macrophages and RA fibroblast-like synoviocytes (FLS) [15,16], and to induce changes in HIF-1α levels and/or transcriptional activation in a number of cell types [17,18]. HIF-1α stabilisation by Th1 cytokines has been demonstrated to be partially mediated by the NFκB and p38 mitogen-activated protein kinase signalling pathways in human articular chondrocytes [19], and by phosphatidylinositol 3-kinase/Akt and MEK1/2 inhibitor activation in RA FLS [16,20]. Importantly, Th1 cytokines have been demonstrated to synergise with hypoxia to induce HIF-1 protein and activity in HepG2 cells, a human hepatoma cell line [18], and to synergise with hypoxia and the hypoxia mimetic CoCl_2 _to induce HIF-1α mRNA and protein, respectively, in RA FLS [16,21]. Inflammatory cytokines and hypoxia were also shown able to act together to augment hypoxia-mediated upregulation of VEGF secretion in RA FLS [22]. As the composition of the RA synovium includes elevated levels of inflammatory cytokines on a hypoxic background, a strong and continuous presence of HIF transcription factors is favoured through increased HIF-α mRNA levels and protein stabilisation. HIFs thus represent a key convergence point that integrates the cellular response of the RA synovium to low oxygen tension and inflammatory cytokines, and thereby drives synovial inflammation and angiogenesis.

Th1 and T-helper cell 2 (Th2) cytokines were recently demonstrated to have differential effects on HIF isoforms. For instance, in macrophages Th1 cytokines induce HIF-1α and Th2 cytokines induce HIF-2α mRNA, and this differential regulation of HIF-1α versus HIF-2α acts to respectively either increase or suppress nitric oxide synthesis and thus to control overall nitric oxide availability [15]. In contrast to Th1 cytokines, anti-inflammatory Th2 cytokines are found at very low levels in RA joints and synovial fluid but they may hold an important therapeutic role in RA via HIFs. For instance IL-4, the signature cytokine of CD4^+ ^Th2 cells, is known to reduce the production of Th1 cytokines by RA synovium [23] - and IL-4 has been used *in vivo *as a treatment for a number of experimental autoimmune diseases in animals, including collagen-induced arthritis (CIA) [24]. By suppressing Th1 cytokine levels, IL-4 may indirectly lower HIF activation and hence the degree of synovial angiogenesis. Despite these interesting aspects, the downstream effects of stimulating RA synovial cells with Th1 versus Th2 cytokines in a hypoxic environment have not yet been investigated with regard to HIF regulation and downstream angiogenic gene expression.

The use of antibodies to cytokines as pharmacological antagonists has revealed the profound effects of anti-TNFα treatment in reducing inflammation and joint destruction in RA. Despite the clear efficacy of anti-TNFα therapy, the actual mechanisms by which TNF-blocking agents are able to obtain these effects are still incompletely understood. In clinical trials using the TNFα inhibitor infliximab, reduced synovial angiogenesis and vascularity appears to be an effect associated with the neutralisation of TNFα [10]. As HIFs regulate a plethora of downstream angiogenic factors including VEGF, in response to hypoxia and inflammatory cytokines, the efficacy of anti-TNFα therapy could, at least in part, be due to a reduction in the activation of HIF leading to decreased angiogenesis and less immature synovial vessels in RA patients.

In the present study, we chose to examine the impact of combined Th1 or Th2 cytokines and hypoxia on the angiogenic signature of RA FLS, focusing particularly on the role of HIF isoforms in the expression of downstream angiogenic genes. RA FLS was the chosen study target due to its recognised role in RA pathogenesis, where it contributes to bone and cartilage breakdown through the acquisition of what appears to be a transformed phenotype [4,25]. Moreover, as this cell type makes up the bulk of the expanding RA synovial membrane tissue [26], it is likely to be exposed to various degrees of hypoxia and inflammatory cytokines simultaneously. We investigated the effect of Th2 cytokines on the angiogenic signature of RA FLS in normoxia and hypoxia in an attempt to elucidate the potential that Th2 cytokines could ameliorate autoimmune disease by influencing angiogenesis.

## Materials and methods

### Isolation and culture of cells from human RA tissue

Total RA synovial membrane cells and FLS were derived from synovial membranes of patients at the Royal Free Hospital (London, UK) who met the American College of Rheumatology 1987 criteria for RA [27]. Full ethical approval was granted for the project (Local Ethics Research Committee EC2003-64). Preoperative informed consent was obtained in all cases.

The RA cell cultures were isolated and cultured as previously published [28,29]. The disaggregated total synovial membrane cell cultures were used directly in experiments. Alternatively, after overnight incubation, nonadherent cells were removed to allow overgrowth of FLS. The purity of the FLS culture was confirmed at passage 3 by immunohistochemistry with monoclonal anti-human-Fibroblast-Surface Protein 1 antibody (Abcam, Cambridge, UK) and determined to be >98%. FLS and normal human skin fibroblasts (HSF; Lonza, Walkersville, MD, USA) were cultured in DMEM containing 10% foetal bovine serum, 4.5 g/l glucose and L-glutamine, supplemented with 100 U/ml penicillin and 100 μg/ml streptomycin (PAA Laboratories, Coelbe, Germany). RA FLS were used between the third and sixth passages.

### Experimental setup and siRNA transfection

RA FLS cell cultures were starved in serum-free DMEM overnight (total synovial membrane cell cultures were used directly in complete medium) and subsequently subjected to a hypoxic gas mixture (1% oxygen, 5% CO_2_, 94% N_2_) for the desired length of time. Alternatively, 1 mM dimethyloxalylglycine (DMOG; Biomol International, Exeter, UK) or dimethyl sulfoxide (DMSO) (as vehicle) or cytokines IL-4, IL-1β, IFNγ (Peprotech, London, UK), TNFα (R&D Systems, Minneapolis, MN, USA) and IL-13 (Abcam) at 10 ng/ml were used to stimulate cells. Total RNA was isolated and the corresponding cDNA was used for quantitative PCR analysis. The cell supernatants were collected for ELISA and for testing in a functional angiogenesis assay.

Knockdown of genes was performed with siRNA against HIF-1α (5'-(AGCAGGUAGGAAUUGGAACAUU)RNA(tt)DNA-3') and/or HIF-2α (5'-(GCGACAGCUGGAGUAUGAAUU)RNA(tt)DNA-3') at a final concentration of 10 nM (MWG, Ebersberg, Germany) in Opti-MEM I Reduced Serum Medium (Invitrogen, Paisley, UK) using Lipofectamine 2000 (Invitrogen). An siRNA oligonucleotide against luciferase mRNA (siLuc) was used as a negative control, as well as oligonucleotides with the scrambled sequence of siHIF-1α or siHIF-2α (data not shown).

### RNA isolation and quantitative PCR

RNA was isolated using the Total RNA E.Z.N.A™ EaZy Nucleic Acid Isolation kit (VWR, Batavia, IL, USA) and was DNase treated (Ambion Ltd, Paisley, UK). First-strand cDNA was synthesised using random primers (Invitrogen) and Moloney Murine Leukaemia Virus reverse transcriptase (Promega, Southampton, UK).

Diluted cDNA was added to SYBR^®^Green I JumpStart™ Taq Ready MIX™ (Sigma-Aldrich, Poole, UK), primer mix and nuclease-free water. Exon-spanning PCR primers (MWG) were designed using Primer 3 (Table 1). All primers were validated prior to use. Primers for the housekeeping gene 18S ribosomal RNA and acidic ribosomal protein (data not shown) were used to normalise samples.

**Table 1:** Primers used in the study

Primer	Forward	Reverse
ANGPTL-4	5´-CCACTTGGGACCAGGATCAC-3´	5´-CGGAAGTACTGGCCGTTGAG-3´
Leptin	5´-GGCTTTGGCCCTATCTTTTC-3´	5´-GGAATGAAGTCCAAACCGGTG-3´
VEGF	5´-CTTGCCTTGCTGCTCTACCT-3´	5´-CTGCATGGTGATGTTGGACT-3´
EFNA3	5´-CACTCTCCCCCAGTTCACCAT-3´	5´-CGCTGATGCTCTTCTCAAGCT-3´
HIF-α	5´-CACCTCTGGACTTGCCTTTC-3´	5´-GGCTGCATCTCGAGACTTTT-3´
HIF-2α	5´-CCTTCAAGACAAGGTCTGCA-3´	5´-TTCATCCGTTTCCACATCAA-3´
18S ribosomal RNA	5´-GTAACCCGTTGAACCCCA-3´	5´-CCATCCAATCGGTAGTAGCG-3´

ANGPTL-4, angiopoietin-like-4; EFNA3, ephrin A3; HIF, hypoxia-inducible factor; VEGF, vascular endothelial growth factor.

Quantitative PCR was performed with the following programme: pre-incubation at 50°C for 2 minutes, initial denaturation at 95°C for 5 minutes, 40 cycles of denaturation at 95°C for 10 seconds, annealing at 60°C for 30 seconds, elongation at 72°C for 30 seconds, and a 20-second final extension step. Rotor-Gene Software version 6.0 was used to analyse the data (Qiagen, Crawley, UK). The comparative cycle threshold (Ct) method (2^-ΔΔCt ^model) was used to calculate relative fold-changes in gene expression [30].

### PCR array

Human Angiogenesis RT^2 ^Profiler™ PCR Arrays (TebuBio, Peterborough, UK) were used to screen cDNA from human RA FLS exposed to 21% or 1% oxygen and to DMSO or 1 mM DMOG. The PCR array was used according to manufacturers' protocol on an ABI 7700 sequence detector (Applied Biosystems, Foster City, CA, USA) using SYBR green technology (TebuBio). Every sample was run on duplicate PCR array plates and the relative quantification of mRNA was performed using the 2^-ΔΔCt ^model and normalised to the average of two or more housekeeping genes on the PCR array.

### Protein measurement by ELISA

Leptin and ANGPTL-4 released into the medium by RA FLS were measured using ELISA duosets (R&D Systems). VEGF was measured using reagents from Becton Dickinson (Oxford, UK) and antibodies from R&D Systems.

### Western blotting

Total protein extracts were separated on NuPAGE Novex Tris-Acetate pre-cast gels 3 to 8% (Invitrogen) under reducing conditions and proteins were blotted onto polyvinylidene fluoride membranes (Perkin Elmer, Waltham, MA, USA). The membranes were blocked with 0.01% Tween 20, PBS and 5% nonfat milk for 1 hour at room temperature, washed and incubated for 2 hours with either anti-human HIF-1α mouse mAb at 1:250 (BD Transduction Laboratories, Oxford, UK) or anti-human α-tubulin mouse mAb at 1:10,000 (Sigma-Aldrich). The membranes were washed and incubated for 1 hour at room temperature with horseradish peroxidase-coupled rabbit anti-mouse IgG at 1:5,000 (Dakocytomation, Glostrup, Denmark), and developed using ECL plus and hyper-film ECL (GE Healthcare, Chalfont St Giles, UK).

### Nuclear extraction and measurement of HIF-1 DNA binding activity

HIF-1 DNA binding activity was determined in RA FLS nuclear extracts. Nuclear extraction was performed according to the manufacturer's protocol (Active Motif, Carlsbad, CA, USA) and the protein concentration was determined using the BCA method. To measure HIF-1 DNA binding activity following stimulation of cells with low oxygen levels and/or cytokines, the nuclear extracts were tested using the ELISA-based TransAM HIF-1 Transcription Factor Assay (Active Motif). A secondary horseradish peroxidase-conjugated antibody and enzyme substrate included in the assay kit were used before HIF-1 binding to HRE was measured by absorbance at 450 nm. Each sample to be assayed for HIF-1 DNA binding was tested in duplicate. The specificity of HIF-1 binding to the HRE-coated wells was confirmed by competition experiments where either wild type or mutated HRE oligonucleotides (Active Motif) were added to the wells along with the nuclear extracts to be tested (data not shown).

### Matrigel tube formation assay

This assay was performed in a 96-well plate with 50 µl Growth Factor Reduced Matrigel per well (VWR, Lutterworth, UK) that was left to gel for 45 minutes at 37°C. Human microvascular endothelial cell (HMEC)-1 (Center for Disease Control and Prevention, Atlanta, GA, USA) was cultured in RPMI containing 5% foetal bovine serum and supplemented with 100 U/ml penicillin and 100 μg/ml streptomycin (PAA Laboratories). The cells were used for matrigel assays when 80% confluent without prior starvation. Cells were dispersed with trypsin and 15,000 cells were loaded into each well in 100 µl full-growth medium. Then 100 µl conditioned medium from RA FLS stimulated with cytokines and/or subjected to hypoxia for 24 hours were added per well and the assay was incubated at 37°C for 4 to 6 hours until sufficient tube formation was visible with a microscope. Direct effects of recombinant TNFα and IL-4 were tested by adding 100 μl of 10 ng/ml cytokine in fibroblast medium per well in triplicate. The tubules were fixed with 4% para-formaldehyde (Sigma-Aldrich), washed in PBS and stained for 5 minutes with 50 µl Gram's Crystal Violet (Sigma-Aldrich), and the washing step was then repeated.

Images were captured with a camera (QICAM FAST; QImaging,Surrey, BC, Canada) attached to a microscope (CKX41 Olympus,Southend-on-Sea, UK) and the matrigel assay was scored using AngioSys Image Analysis software (TCS Cell Works, Buckingham, UK). This program provides quantitative measurement of tubule development in images captured from the 96-well plate, analysing the three parameters of total tubule number, number of tubule junctions and percentage of field area covered by tubules. Each experimental condition was tested in triplicate wells with a single picture captured from the centre of each well. The images were blinded prior to analysis.

### Statistical analysis

Data were analysed using Prism software (Graph Pad Software, San Diego, CA, USA). *P *values were determined using a paired two-tailed *t *test assuming unequal variances or where indicated a one-way analysis of variance with Bonferroni's multiple comparison test.

## Results

### PCR arrays identify seven genes as hypoxia-regulated in a HIF-dependent manner in RA FLS

To determine which angiogenic factors are produced by RA FLS in response to hypoxia, we isolated RA FLS from synovial tissue of five patients, cultured them for 4 or 24 hours under either hypoxic conditions (1% oxygen) or normoxic conditions (21% oxygen), and screened the cDNA using a Human Angiogenesis RT^2 ^Profiler™ PCR array. These arrays include 84 genes that have all been implicated in the process of angiogenesis in various cell types and settings. (See Additional file 1 for a table showing as an example the results from the screening of hypoxic FLS from one RA patient.

**Figure 1:**
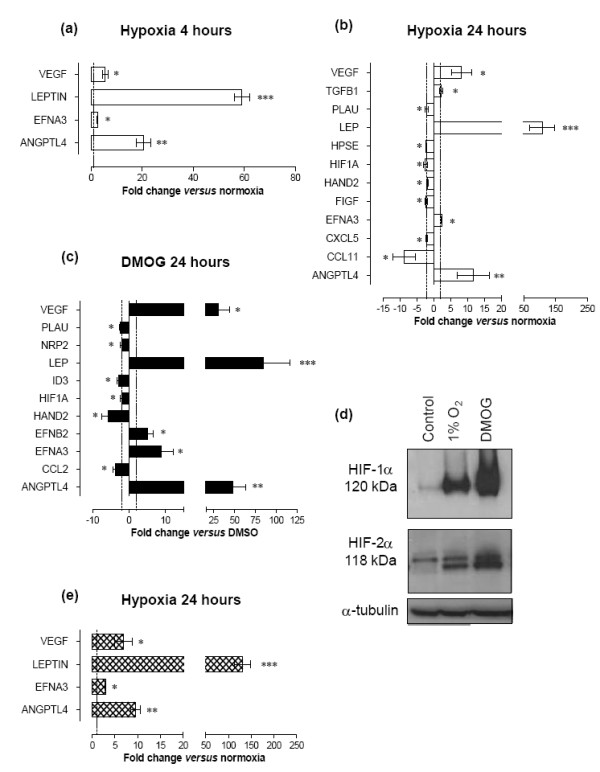
**Angiogenesis RT² Profiler™ PCR Array data and expression analysis of human rheumatoid arthritis cells**. PCR arrays were used with cDNA from rheumatoid arthritis (RA) fibroblast-like synoviocytes (FLS) from five RA patients and incubated for either **(a) **4 hours or **(b) **24 hours in 1% oxygen (hypoxia) and **(c) **from three RA patients exposed to 1 mM dimethyloxalylglycine (DMOG) for 24 hours. Values are fold-change versus normoxia (21% oxygen) or dimethyl sulfoxide (DMSO) control and were analysed against housekeeping gene β-actin, 18S ribosomal RNA, hypoxanthine phosphoribosyltransferase 1 and 60S ribosomal protein L13a. Genes significantly increased or decreased by a factor ≥2 (*P *< 0.05, dotted line) in all patients are shown. Data are mean ± standard error of the mean and were analysed by paired *t *test of ΔCt values, comparing normoxia versus hypoxia (a and b) or DMSO control versus DMOG (c). **(d) **Western blotting demonstrates induction of hypoxia-inducible factor (HIF)-1α and HIF-2α in total protein lysates from RA FLS in response to 1% oxygen or 1 mM DMOG for 24 hours. An antibody against α-tubulin was used as a loading control. A lane irrelevant to the study was removed as indicated by the line to show lanes of interest adjacent to one another. **(e) **Freshly dissociated total human RA synovial membrane cells from four patients were analysed for expression of vascular endothelial growth factor (VEGF), leptin, ephrin A3 (EFNA3) and angiopoietin-like (ANGPTL)-4 using quantitative PCR. Changes in mRNA are expressed as fold-change relative to levels under 21% oxygen set as 1.0 (dotted line). *P < 0.05, **P < 0.01, ***P < 0.001. EFNB2, ephrin B2; FIGF, C-fos-induced growth factor (VEGF D); HAND2, Heart and neural crest derivatives expressed 2; HPSE, heparanase; ID3, inhibitor of DNA-binding 3, dominant negative helix-loop-helix protein; NRP2, neuropillin 2; PLAU, plasminogen activator urokinase.

Of the 84 known angiogenic genes on the array, the mRNA level of four genes changed after 4 hours - namely ephrin A3 (EFNA3; threefold, *P *< 0.05), VEGF (sixfold, *P *< 0.05), and adipokines angiopoietin-like (ANGPTL)-4 (21-fold, *P *< 0.01) and leptin (59-fold, *P *< 0.001) (Figure 1a). After 24 hours, 12 genes were significantly altered in RA FLS in response to hypoxic exposure from all patients (≥2-fold increase or decrease in all five patients at *P *< 0.05) (Figure 1b). The two genes that were induced most dramatically after 24 hours of hypoxia were leptin (108-fold, *P *< 0.001) and ANGPTL-4 (12-fold, *P *< 0.05). This induction was greater than that observed for VEGF (eightfold, *P *< 0.05), a well-characterised hypoxia-regulated gene. The induction of ANGPTL-4 and leptin by hypoxia is in agreement with a recent microarray expression study in RA FLS [31]. In addition to ANGPTL-4 and leptin, the expression of a receptor protein tyrosine kinase, EFNA3, was also increased by hypoxia (twofold, *P *< 0.01). Furthermore, a significant reduction was seen in the mRNA of HIF-1α, plasminogen activator urokinase, , heart and neural crest derivatives expressed 2, C-fos-induced growth factor (VEGF D), chemokines CCL-11 and CXCL-5, and heparanase (Figure 1b). The largest reduction in gene expression (ninefold, *P *< 0.05) was seen for CCL-11 (which encodes the protein eotaxin, best known for its role in neutrophil chemotaxis).

To establish whether the hypoxia-regulated genes were induced in a HIF-dependent or HIF-independent manner, we stimulated FLS from three RA patients for 24 hours with DMOG, resulting in similar but not identical responses to hypoxia (Figure 1c). DMOG is a nonspecific inhibitor of prolyl hydroxylases leading to both HIF-1α and HIF-2α accumulation as the subunits no longer become hydroxylated and targeted for proteosomal degradation (Figure 1d). DMOG induced changes in the expression of 11 genes, seven of which were also hypoxia responsive (Table 2). These included leptin (85-fold, *P *< 0.001), ANGPTL-4 (48-fold, *P *< 0.01), EFNA3 (ninefold, *P *< 0.05) and VEGF (31-fold, *P *< 0.05), which were upregulated, and HIF-1α (twofold, *P *< 0.05) that was downregulated, implicating HIF transcription factors as regulators of these hypoxia-responsive genes. The HIF-mediated downregulation of HIF-1α mRNA in hypoxic RA FLS confirms the findings of a parallel study (H Larsen, B Muz, M Feldmann, EM Paleolog, unpublished data). In addition, genes that were altered in response to DMOG but not to hypoxia included increased ephrin B2, reduced inhibitor of DNA-binding 3, dominant negative helix-loop-helix protein, neuropillin 2 and CCL-2, demonstrating that the effects of DMOG and hypoxia are not interchangeable.

**Table 2:** Genes significantly upregulated or downregulated by hypoxia and/or dimethyloxalylglycine in human rheumatoid arthritis fibroblast-like synoviocytes

Genes that change only in response to hypoxia	Genes that change only in response to DMOG	Genes that change in response to hypoxia and DMOG
TGF-β_1 _(2*)	NRP2 (-2*)	VEGF (8*/31*)
HPSE (-2*)	ID3 (-3*)	PLAU (-2*/-2*)
FIGF (-2*)	EFNB2 (5*)	LEP (108***/85***)
CXCL-5 (-2*)	CCL2 (-4*)	HIF1A (-2*/-2*)
CCL-11 (-9*)		HAND2 (-2*/-6*)
		EFNA3 (2*/9*)
		ANGPTL4 (12**/48**)

Genes significantly upregulated or downregulated by a factor ≥2. Genes from Figure 1b,c that change following 24 hours of hypoxia and in response to stimulation with the hypoxia-inducible factor activator dimethyloxalylglycine (DMOG) in rheumatoid arthritis fibroblast-like synoviocytes from five and three patients, respectively. Data presented as mean fold-change. Genes that changed significantly (*P *< 0.05) by a factor ≥2-fold were analysed by paired Student's *t *test comparing ΔCt values: *P < 0.05, **P < 0.001, ***P < 0.001. ANGPTL, angiopoietin-like; EFNA3, ephrin A3;EFNB2, ephrin B2; FIGF, C-fos-induced growth factor (VEGF D); HAND2, Heart and neural crest derivatives expressed 2; HPSE, heparanase; ID3, inhibitor of DNA-binding 3, dominant negative helix-loop-helix protein; LEP, leptin; NRP2, neuropillin 2; PLAU, plasminogen activator urokinase; TGF, transforming growth factor; VEGF, vascular endothelial growth factor.

As the constituents of RA joints are invariably of mixed cell types including FLS as well as T cells, B cells, endothelial cells and macrophages, we tested the relevance of our findings by subjecting total cell isolates from synovial membranes of RA patients to 1% oxygen for 24 hours to compare data from RA FLS. Gene expressions for leptin, ANGPTL-4, EFNA3 and VEGF were significantly upregulated (132-fold, 10-fold, threefold and sevenfold increase, respectively) following 24 hours of stimulation with hypoxia, mirroring responses obtained from RA FLS alone (Figure 1e). Furthermore, we exposed FLS to a range of oxygen tensions (1 to 10% oxygen) and found that the genes of interest (ANGPTL-4, VEGF, leptin, EFNA3) change in a similar fashion at 3%, the median oxygen tension measured in RA synovium when compared with 1% oxygen, albeit to a lesser extent (see Additional file 2). At 10% oxygen, which is the median oxygen tension found in healthy synovium, we no longer observed any effect on angiogenic gene expression.

### Hypoxia induces leptin, ANGPTL-4 and ephrin A3 in a HIF isoform-dependent manner

As leptin, ANGPTL-4 and EFNA3 were the genes that were upregulated to the greatest extent on the array by hypoxia and also by the HIF activator DMOG, these genes were selected for further investigation. Importantly, we wanted to elucidate which of the two HIF isoforms, HIF-1 or HIF-2, might be involved in the induction of these genes. Leptin and ANGPTL-4 have previously been reported to be regulated by hypoxia in a HIF-1-dependent manner in adipocytes, human skin fibroblasts, HeLa cells, and MCF-7 breast cancer cells (leptin) [32-34], and in cadiomyocytes (ANGPTL-4) [35], but none of these studies addressed the involvement of the HIF-2 isoform. Induction of EFNA3 by HIFs has not previously been investigated. To examine the relative importance of either HIF isoform in inducing angiogenic responses, we used siRNA technology to knock down HIF-1α and HIF-2α individually or in combination. The efficiency of HIF knockdown using siRNA oligonucleotides was confirmed at the mRNA level using quantitative PCR, with HIF-1 reduced by 74% using siHIF-1 (*P *< 0.001 versus siLuc) and 80% for HIF-2 using siHIF-2 (*P *< 0.001 versus siLuc), and at the protein level by Wblotting (see Additional file 2).

**Figure 2:**
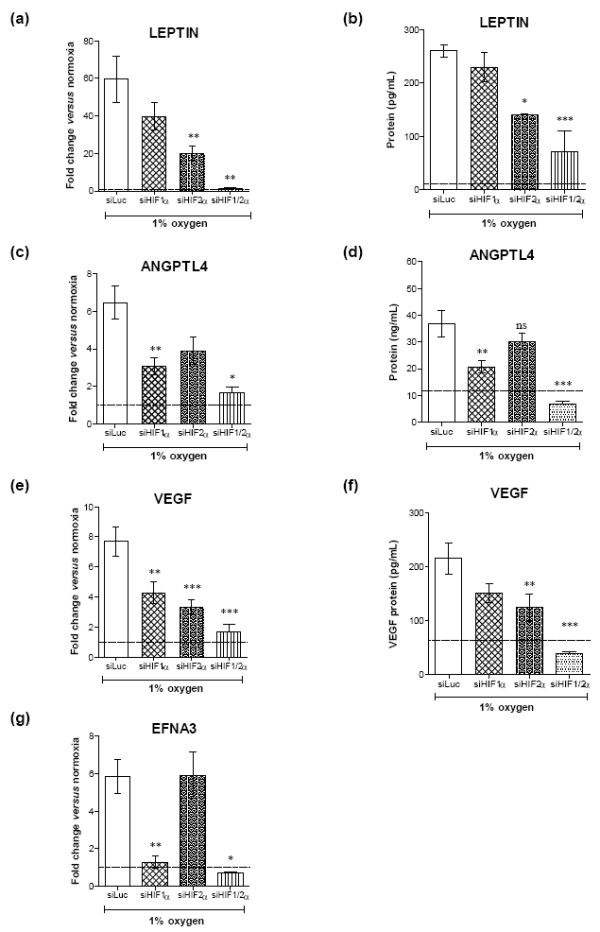
**Hypoxia-induced hypoxia-inducible factor isoform dependence of angiogenic genes in human rheumatoid arthritis fibroblast-like synoviocytes**. Hypoxia-induced hypoxia-inducible factor (HIF) isoform dependence of ephrin A3 (EFNA3), angiopoietin-like (ANGPTL)-4, leptin and vascular endothelial growth factor (VEGF) in human rheumatoid arthritis (RA) fibroblast-like synoviocytes (FLS). RA FLS were transiently transfected with siRNA oligonucleotides complementary to hypoxia-inducible factor (HIF)-1α (siHIF-1α) or HIF-2α (siHIF-2α) or both simultaneously. An siRNA oligonucleotide complementary to siLuc was used as control. The cell cultures were subsequently exposed to 1% oxygen (hypoxia) for 24 hours. Total RNA was isolated and cDNA generated, and the mRNA level of **(a) **leptin and **(c) **ANGPTL-4, **(e) **VEGF and **(g) **EFNA3 was determined using quantitative PCR. Changes in mRNA expressed as fold-change relative to levels in the siLuc transfected normoxic controls set as 1.0 (dotted line). The secretion of **(b) **leptin, **(d) **ANGPTL-4 and **(f) **VEGF protein was measured using ELISA. Data expressed as mean ± standard error of the mean of ≥3 independent experiments with sample assayed in triplicate, and were analysed using one-way analysis of variance with Bonferroni's *post-hoc *test for multiple comparisons versus hypoxic siLuc transfected cells (*P < 0.05, **P < 0.01, ***P < 0.001).

We found hypoxia-induced leptin to be predominantly dependent on HIF-2 as knockdown of HIF-2α resulted in 66% reduction in leptin expression relative to siLuc, while the effect of siHIF-1α was more modest (33% reduction in leptin expression; Figure 2a). Addition of a combination of siHIF-1α and siHIF-2α almost completely abolished hypoxia-induced leptin expression, demonstrating the involvement of both isoforms (98% reduction relative to siLuc). In contrast, HIF-1 and HIF-2 appeared to contribute more evenly to ANGPTL-4 induction, as HIF-1α or HIF-2α knock down resulted in reduction of ANGPTL-4 expression by 52% and 40%, respectively, relative to cells transfected with siLuc (Figure 2c). Accordingly, a combination of siHIF-1α and siHIF-2α had an even greater knockdown effect on ANGPTL-4 expression of 75% compared with siLuc transfected cells. VEGF expression was also dependent on both HIF isoforms (Figure 2e); however, knock down of HIF-2α seemed to have a slightly stronger impact on the VEGF transcript level (57% reduction) than knock down of HIF-1α (44% reduction). Knock down of both HIF isoforms reduced VEGF expression even further (78% reduction). Interestingly, EFNA3 appeared to be regulated solely by HIF-1 (78% reduction in EFNA3 expression by siHIF-1α relative to siLuc), where HIF-2α knockdown was without effect on the hypoxia-induced transcript level (Figure 2g). Hence the double HIF isoform knockdown had no further effect compared with HIF-1α knockdown. The HIF-isoform dependence in hypoxia-treated cells was confirmed at the protein level for leptin, ANGPTL-4 and VEGF, and gave rise to similar results (Figure 2b,d,f). Western blotting for EFNA3 was unsuccessful - probably due to the protein being simultaneously downregulated in hypoxia, which was previously demonstrated in human umbilical vein endothelial cells [36].

### Th1 cytokines specifically drive a HIF-1 isoform response by RA FLS

As demonstrated above, hypoxia stabilises HIF-1α and HIF-2α protein subunits, thereby enabling the formation of active HIF transcription factors and subsequent production of angiogenic factors. As the RA synovial tissue in patients is characterised by elevated levels of inflammatory Th1 cytokines in addition to hypoxia, we next investigated the effect of a selection of Th1 and Th2 cytokines, relevant to RA disease, on HIF-1 and HIF-2 in RA FLS. Cytokines have previously been shown to induce HIFs, and selective induction of HIF-1 by Th1 cytokines and of HIF-2 by Th2 cytokines has been demonstrated in macrophages [15] but has not been investigated in cells relevant to RA. In particular, we examined the effect on HIFs of combining Th1 or Th2 cytokine stimulation with 1% oxygen on HIF activity and HIFα protein levels.

**Figure 3:**
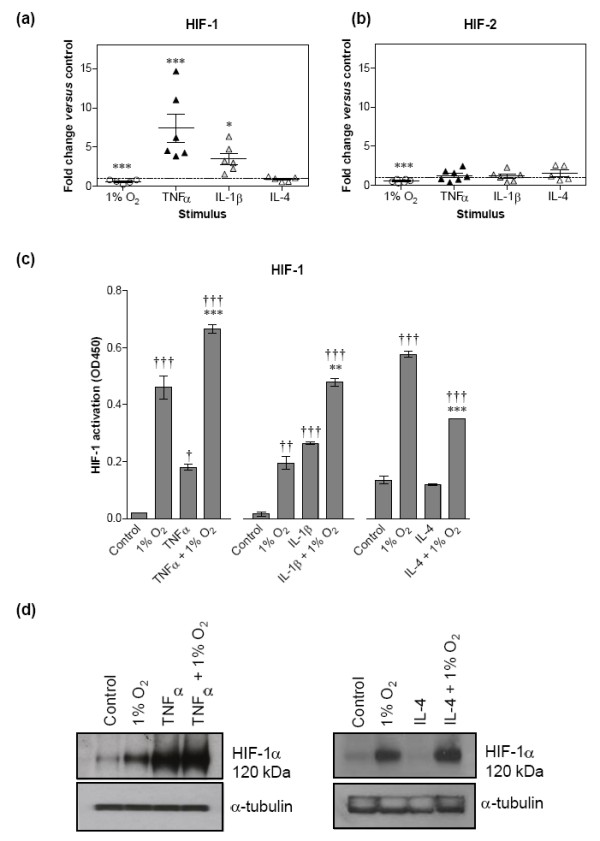
**Th1 cytokines induce hypoxia-inducible factor-1 mRNA, protein and activity whereas Th2 cytokines have no effect**. Cell cultures were exposed to 10 ng/ml cytokine, 1% oxygen, or left untreated for 24 hours. Total RNA was isolated and cDNA generated, and the mRNA level of **(a) **hypoxia-inducible factor (HIF)-1α and **(b) **HIF-2α was determined using quantitative PCR. Changes in mRNA levels expressed as fold-change relative to levels in untreated controls (dotted line). Data are mean ± standard error of the mean and were analysed by paired *t *test of ΔCt values versus control (*P < 0.05, ***P < 0.001). **(c) **HIF-1 binding to wells pre-coated with human hypoxia-response element oligonucleotides in response to 1% oxygen (hypoxia) and cytokine stimulation, alone or in combination, was measured using nuclear extracts and a TransAm HIF-1 DNA binding kit. DNA binding activity is expressed as the optical density at 450 nm and represents the mean ± standard deviation from three separate representative experiments. Samples were analysed using one-way analysis of variance with Bonferroni's *post-hoc *test for multiple comparisons versus control (†*P *< 0.05, ††*P *< 0.01, †††*P *< 0.001) or versus hypoxia alone (**P < 0.01, ***P < 0.001). **(d) **Western blots showing HIF-1α protein levels in response to 1% oxygen, TNFα, IL-4 and cytokines in combination with 1% oxygen for 24 hours. An antibody against α-tubulin was used as a loading control.

To examine the effect of this combination we stimulated RA FLS with 10 ng/ml of the Th1 cytokines TNFα and IL-1β or the Th2 cytokines IL-4 and IL-13 in 21% or 1% oxygen for 24 hours and investigated changes in gene induction by quantitative PCR, Western blotting and using a HIF DNA binding assay. By drawing comparisons with RA FLS subjected to hypoxia alone, we found that HIF-1α transcript was induced by Th1 cytokines TNFα and IL-1β (on average 7.4-fold and 3.5-fold, respectively), but not by Th2 cytokines IL-4 (Figure 3a) and IL-13 (data not shown). In contrast, HIF-2α mRNA level was unaffected by stimulation with either Th1 or Th2 cytokines (Figure 3b). In contrast, the mRNA of both HIF-α isoforms was reduced by hypoxia by more than 40% (Figure 3a,b), an effect we had also observed on the PCR arrays for HIF-1α (Figure 1b,c). The effect of the cytokines was confirmed at the activity level using a TransAm assay from Active Motif, which measures HIF-1 binding to HRE-coated wells. HIF-1 DNA binding was induced ninefold by TNFα and 16.5-fold by IL-1β, whereas IL-4 exerted a significantly negative effect on HIF-1 binding under hypoxia (Figure 3c). In comparison, HIF-1 DNA binding was induced on average between fourfold and 23-fold by hypoxia alone. IL-13 had no effect on HIF-1α or HIF-2α transcript induction, protein level or HIF DNA binding activity (data not shown). When we stimulated FLS with cytokines in combination with 1% oxygen we found that both Th1 cytokines tested had an additive effect with hypoxia on HIF-1 DNA binding.

Induction of HIF-1α protein by TNFα and lack thereof in response to IL-4 was further shown by Western blotting (Figure 3d). The additive effect of TNFα and hypoxia on HIF-1 binding was also seen at the HIF-1α protein level, demonstrating that HIF-1 serves as a convergence point for hypoxia and inflammatory signalling pathways in RA FLS. Unlike hypoxia that induces both HIF isoforms in RA FLS, Th1 cytokines thus drive activity of HIF-1 only. In contrast to what has been shown in other cell types, the anti-inflammatory Th2 cytokines did not appear to have any positive effect on either of the HIF isoforms in RA FLS, whereas IL-4 had an inhibitory effect on HIF-1 activity. Finally, for comparison we tested the effect of IFNγ, a Th1 cytokine that is not associated with RA, on HIFα mRNA levels in RA FLS. As observed with TNFα and IL-1β, IFNγ induced HIF-1α whereas it had a negative effect on HIF-2α mRNA levels (see Additional file 2), suggesting that RA FLS generally respond to Th1 cytokines by upregulating HIF-1α.

### Th1 cytokines enhance hypoxia-mediated induction of ephrin A3 and VEGF but negatively regulate adipokines ANGPTL-4 and leptin

We established above that only HIF-1 is induced by both hypoxia and RA-associated Th1 cytokines and that Th2 cytokines do not induce HIFs in RA FLS. We next aimed to determine whether Th1 cytokine induction of HIF-1 would induce expression of EFNA3, ANGPTL-4 and leptin similar to that observed in hypoxia. In particular, we wanted to investigate the effect of combined Th1 cytokines and hypoxia on the expression of these genes in RA FLS. We included IL-4 and IL-13 to determine whether Th2 cytokines had any HIF-independent effects on the angiogenic genes investigated.

RA FLS were exposed to 10 ng/ml of either the Th1 cytokines TNFα and IL-1β or the Th2 cytokines IL-4 and IL-13, as single stimuli or in combination with 1% oxygen for 24 hours. As hypoxia and Th1 cytokines both induced HIF-1, our starting hypothesis was that the HIF-1 target genes VEGF, ANGPTL-4 and EFNA3 (and to a lesser degree leptin) would be upregulated by both conditions. Furthermore, we expected the additive effect on HIF-1 DNA binding activity of hypoxia and cytokines in combination would be transferred to induction of the HIF-1 target genes.

**Figure 4:**
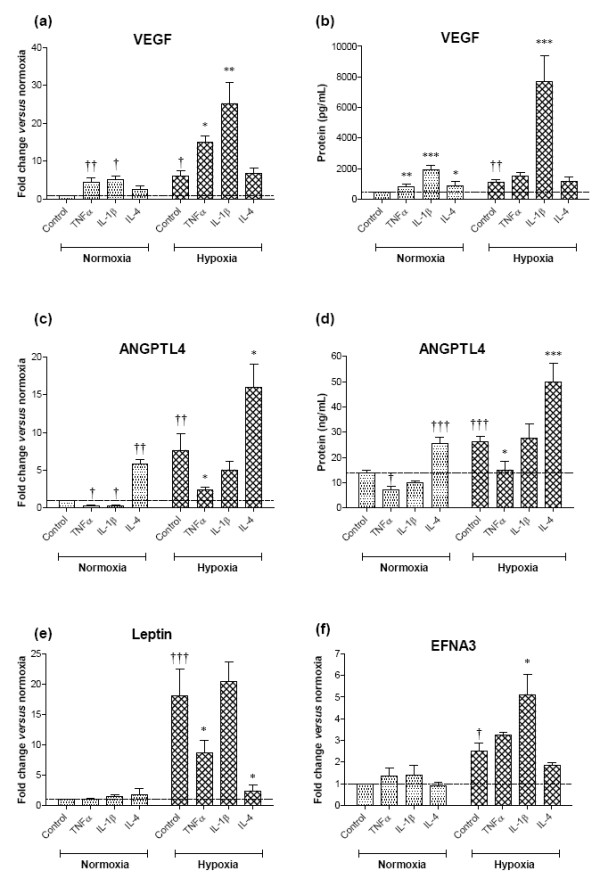
**Proangiogenic/anti-angiogenic effects of Th1 and Th2 cytokines on rheumatoid arthritis fibroblast-like synoviocyte gene expression**. Cell cultures were exposed to 1% oxygen (hypoxia) and/or 10 ng/ml cytokine or left untreated for 24 hours. Total RNA was isolated and cDNA generated, and the mRNA level of **(a) **vascular endothelial growth factor (VEGF), **(c) **angiopoietin-like (ANGPTL)-4, **(e) **leptin and **(f) **ephrin A3 (EFNA3) was determined using quantitative PCR. Changes in mRNA expressed as fold-change relative to levels in untreated samples set as 1.0 (dotted line). Secretion of **(b) **VEGF and **(d) **ANGPTL-4 protein was measured using ELISA. Data expressed as the mean ± standard error of the mean of ≥3 independent experiments with samples assayed in triplicate. Samples analysed using one-way analysis of variance with Bonferroni's *post-hoc *test for multiple comparisons versus control normoxia (†*P *< 0.05, ††*P *< 0.01, †††*P *< 0.001) or versus hypoxia alone (*P < 0.05, **P < 0.01,***P < 0.001).

As expected, VEGF transcripts and protein were induced by both Th1 cytokines IL-1β and TNFα, and the observed effects were additive (TNFα) or even synergistic (IL-1β) compared with hypoxia alone (Figure 4a,b) as was previously reported [22]. Furthermore, both Th1 cytokines had a modest effect on EFNA3 expression in normoxia, with IL-1β interacting with hypoxia to generate an additive effect upon EFNA3 expression whereas combined TNFα with hypoxia had no further effect (Figure 4f). In contrast to what we observed for VEGF and EFNA3, the Th1 cytokines TNFα and IL-1β had strong negative effects on ANGPTL-4 expression both as single stimuli and in combination with hypoxia (Figure 4c,d), despite the ability of Th1 cytokines to induce HIF-1 activity. TNFα also had an inhibitory effect on leptin under hypoxic conditions, whereas IL-1β had no effect on leptin expression (Figure 4e).

**Figure 5:**
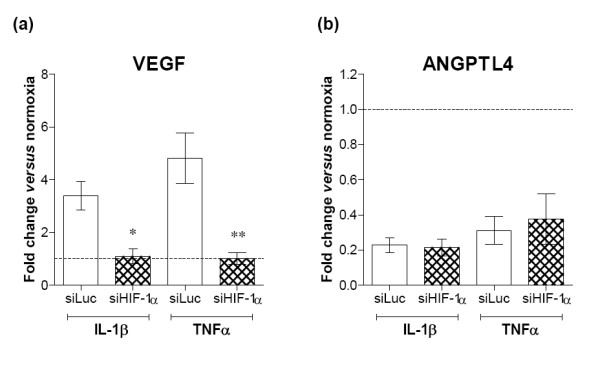
**Th1-induced vascular endothelial growth factor but not angiopoietin-like-4 expression is dependent on hypoxia-inducible factor-1**. Rheumatoid arthritis (RA) fibroblast-like synoviocytes (FLS) were transiently transfected with siRNA oligonucleotides complementary to hypoxia-inducible factor (HIF)-1α (siHIF-1α). An siRNA oligonucleotide complementary to siLuc was used as control. The cell cultures were subsequently exposed to 10 ng/ml cytokines or left untreated for 24 hours. Changes in mRNA in response to IL-1β or TNFα of **(a) **vascular endothelial growth factor (VEGF) and **(b) **angiopoietin-like (ANGPTL)-4 expressed as fold-change relative to levels in the siLuc transfected untreated controls set as 1.0 (dotted line). Data expressed as the mean ± standard error of the mean of ≥3 independent experiments with samples assayed in triplicate and analysed versus cytokine-treated siLuc transfected cells. *P < 0.05, **P < 0.01.

In contrast to the Th1 cytokines, IL-4 stimulation of RA FLS led to a strong induction of ANGPTL-4 expression of similar magnitude to that observed by hypoxic stimulation alone. This effect was greatly amplified in the presence of hypoxia and observed at both the mRNA and protein levels (Figure 4c,d). IL-4 induced VEGF mRNA production and protein secretion by the cells (Figure 4a,b), whereas this cytokine had no effect on expression of EFNA3 (Figure 4f). IL-13 had no effect on angiogenic gene expression under normoxia or hypoxia (data not shown). Interestingly, IL-4 also had anti-angiogenic effects because it could almost completely abrogate hypoxia-induced leptin (Figure 4e). Since we had already established that HIFs are activated by Th1 cytokines and not by Th2 cytokines, the observed effects of IL-4 on ANGPTL-4 and VEGF are HIF independent. As was expected, we found Th1 cytokine-mediated induction of VEGF to be mediated by HIF-1, which we demonstrated using our siRNA oligonucleotides against the HIF isoforms (Figure 5a). The negative effects of Th1 cytokines on ANGPTL-4 expression by normoxic RA FLS was independent of HIF-1, as demonstrated by HIF-1α isoform knock down that did not restore basal ANGPTL-4 expression (Figure 5b). There thus seems to be a differential regulation of HIF-1 target genes by Th1 cytokines and hypoxia, although both stimuli induce HIF-1.

We also examined whether the negative effect of Th1 cytokines on adipokine expression was specific to RA FLS, and therefore RA disease related, by comparing our findings with those obtained with normal HSF. TNFα had a similar negative effect on leptin expression in both cell types (see Additional file 2). In contrast, the negative effect of TNFα on ANGPTL-4 previously observed in RA FLS was absent in HSF, where a gradual increase in ANGPTL-4 mRNA was observed with stimulation time (see Additional file 2), suggesting that ANGPTL-4 inhibition by TNFα is a pathological feature of RA FLS.

### Differential effects of Th1 and Th2 cytokines on HMEC-1 tubule formation in *vitro*

**Figure 6:**
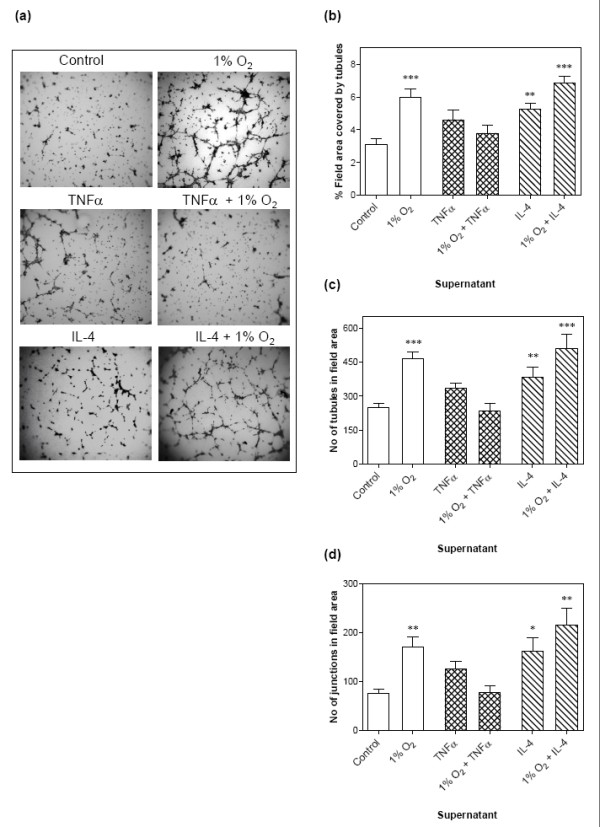
**Interactions of hypoxia and cytokines modulate induction of angiogenic activity by rheumatoid arthritis fibroblast-like synoviocytes**. Rheumatoid arthritis (RA) fibroblast-like synoviocytes (FLS) were cultured in normoxia (21% oxygen), hypoxia (1% oxygen) or with 10 ng/ml IL-4 or TNFα for 24 hours. Alternatively the cells were co-stimulated with cytokines whilst cultured in hypoxia to mimic the environment in RA joints. Supernatants from these cultures were applied to wells of a 96-well plate containing growth factor-reduced matrigel and pre-seeded human microvascular endothelial cell (HMEC)-1 cells and left to form tubules for 4 to 6 hours. **(a) **A single picture was captured from the centre of each well with a camera (QICAM FAST; QImaging) attached to a microscope (CKX41; Olympus), with a representative example for each condition shown here. Using AngioSys Image Analysis software we analysed several parameters of angiogenesis: **(b) **percentage of field area covered by HMEC-1 tubules, **(c) **number of tubules, and **(d) **tubule junctions formed in the field area. Data expressed as the mean ± standard error of the mean of ≥3 independent experiments with supernatants from each condition assayed in triplicate wells. Samples analysed using one-way analysis of variance with Bonferroni's *post-hoc *test for multiple comparisons versus control (*P < 0.05, **P < 0.01, ***P < 0.001).

As demonstrated above, cytokines in combination with hypoxia activate complex HIF-dependent and HIF-independent responses by RA FLS. The eventual outcome of tissue neovascularisation is dependent upon a delicate balance between positive and negative regulators of angiogenesis. To elucidate the functional significance of the uncovered angiogenic gene signatures following cytokine and hypoxia stimulation in RA FLS, we took FLS supernatants and applied these in an *in vitro *angiogenesis assay. Conditioned media from hypoxic RA FLS had a marked proangiogenic effect on the endothelial cells compared with supernatants from control RA FLS (Figure 6a) and increased both the percentage of the total field area covered by tubule-like structures (Figure 6b), as well as the number of tubules (Figure 6c) and tubule junctions (Figure 6d). The conditioned medium from RA FLS pretreated with 10 ng/ml IL-4 for 24 hours also induced angiogenesis, albeit to a lesser extent than hypoxia (Figure 6a to d). We next investigated the angiogenic drive of conditioned medium from cells co-stimulated with IL-4 and hypoxia for 24 hours. We observed this medium to be more angiogenic than supernatants from cells subjected to either condition alone (Figure 6a), with regard to both the increased percentage of total field area covered by tubules as well as the number of tubules and tubule junctions (Figure 6b to 6d).

The conditioned medium from RA FLS stimulated with 10 ng/ml TNFα alone induced a modest angiogenic response with HMEC-1 cells. Interestingly, the proangiogenic effect observed for hypoxia was absent when supernatants derived from cells exposed to TNFα and hypoxia simultaneously were applied (Figure 6a to 6d). These supernatants did not exhibit an increased angiogenic effect over that of supernatants from unstimulated RA FLS despite a much higher VEGF protein content (Figure 4b).

We also tested the direct effects of recombinant TNFα and IL-4 on tubule formation by the endothelial cells. Both induced tubule formation in the matrigel assay (data not shown). Although unlikely, unbound recombinant cytokine in the normoxic supernatants could account for the low angiogenic activity observed. This cannot, however, explain the reduction in tubule formation seen by supernatants from hypoxic RA FLS co-stimulated with TNFα, nor can it explain the larger angiogenic effect observed with supernatants from hypoxic RA FLS stimulated with IL-4.

## Discussion

Angiogenesis in RA is an important hallmark of disease, and the degree of angiogenesis and immature blood vessel formation correlates with inflammation and disease activity. Interestingly, therapies targeting Th1 cytokines (for example, TNFα and IL-1β) have been shown to reduce angiogenesis in addition to decreasing inflammation, whereas therapies using Th2 cytokines ameliorate RA disease through less well described mechanisms [6,24]. The aim of the present work was therefore to elucidate how Th1 and Th2 cytokines affect angiogenesis in RA, alone and in combination with hypoxia, a major driving force for angiogenesis in RA. Such research can contribute to the understanding of how therapies directed against Th1 cytokines directly and indirectly affect angiogenesis, and also whether Th2 cytokines reduce disease severity through an effect on angiogenesis. Moreover, understanding how angiogenic factors are affected by inflammatory and anti-inflammatory cytokines against a background of synovial hypoxia may pave the way for future optimisation of existing RA therapies to better target the dual aspect of RA disease, namely angiogenesis and inflammation.

We specifically chose to focus on the differential effects of Th1 versus Th2 cytokines and hypoxia on HIFs, the master regulators of angiogenesis, and on the expression of downstream angiogenic genes by RA FLS. FLS are well-described contributors to inflammation and joint degradation in RA [4,25]. As hypoxia and proinflammatory cytokines typically co-exist within the RA synovium, we were particularly interested in examining the angiogenic effect of combined stimuli on RA FLS given that both types of stimuli are capable of inducing HIF expression [16]. Although we demonstrated by PCR arrays that several other novel angiogenic genes were significantly altered in RA FLS exposed to hypoxia and the HIF activator DMOG, we focused on the genes EFNA3, leptin, ANGPTL-4 and VEGF as surrogate markers of HIF-induced genes and angiogenesis because they demonstrated consistent and maximal induction on human angiogenesis PCR arrays following hypoxia and DMOG stimulation. The first gene, EFNA3, is a component of the Eph/ephrin tyrosine kinase system and this receptor/ligand system is associated with various signalling pathways related to cell growth and viability, cytoskeletal organisation, cell migration, and apoptosis (reviewed in [37]). In adult life, ephrin upregulation - particularly that of ephrin B - has been correlated to vascular invasion, blood vessel formation and sprouting by tumours, and soluble Eph A receptors have been shown to inhibit tumour angiogenesis [38]. EFNA3 has not previously been associated with RA.

The adipokines, leptin and ANGPTL-4, are secreted mainly by the liver and adipocytes, and their original role as primary energy regulators in cell metabolism has progressively encompassed other functions including modulation of immune and inflammatory processes as well as angiogenesis [39,40]. Serum leptin levels have been reported to be elevated in RA patients and to correlate to accelerated atherosclerosis, thus potentially accounting for increased incidence of cardiovascular disease afflicting RA patients [41,42]. ANGPTL-4 has likewise been linked with arthritis because it was identified in a gene expression profiling analysis as one of the most highly expressed genes in early CIA, a widely used mouse model of RA. Moreover, ANGPTL-4 transcript has been reported to localise to stromal fibroblast-like cells adjacent to blood vessels in the mouse arthritic tissue, confirming that - like human RA FLS - murine RA FLS also secrete ANGPTL-4 [43].

Although ANGPTL-4 and leptin have previously been reported to be upregulated by hypoxia in RA FLS, the study by Del Rey and colleagues did not investigate the involvement of HIFs or cytokines in adipokine expression by RA FLS [31]. We demonstrated for the first time that these hypoxia-regulated genes were differentially regulated by HIF isoforms under hypoxic conditions, with leptin being primarily HIF-2 dependent, whereas ANGPTL-4 and VEGF were both HIF-1 and HIF-2 dependent, and EFNA3 was induced mainly by HIF-1. We have recently shown that these genes are similarly regulated in osteoarthritis and RA FLS by HIFs and prolyl hydroxylase-2, but not in normal HSF, suggesting that this is a pathological feature of synoviocytes from both diseases [44]. Once we had established the relative contribution of HIF isoforms to the hypoxic induction of the adipokines and EFNA3 in RA FLS, we proceeded to investigate the regulation of HIFs by cytokines alone and in combination with hypoxia, as has been done before for HIF-1 and Th1 cytokines [21] but not for Th2 cytokines and HIF-2. Th1 and Th2 cytokines were previously reported to have differential effects on HIF isoforms in macrophages, with Th1 cytokines inducing HIF-1 and Th2 cytokines inducing HIF-2 [15], but a similar relationship has not been established in RA FLS. We demonstrated that HIF-1 was activated by Th1 cytokines, but not when RA FLS were stimulated with Th2 cytokines. In contrast, HIF-2 expression was unaffected by cytokine stimulation of either kind, thus highlighting a redundant role of HIF-2 in mediating cytokine-induced angiogenic responses in RA FLS.

Interestingly, unlike the induction by Th1 cytokines, we found that prolonged hypoxia reduced HIF-1α mRNA levels although HIF-1α protein was concomitantly upregulated. The downregulation of HIF-1α in hypoxia has been described before in a human lung epithelial cell line (A549) and is thought to be due to message destabilisation by a naturally occurring antisense to HIF-1α, aHIF [29,45]. aHIF is therefore possibly responsible for hypoxia-mediated downregulation of HIF-1α in RA FLS. HIF-2α mRNA was also downregulated by hypoxia in our study; however, as aHIF is complementary only to the 3'-UTR of HIF-1α but not to any part of HIF-2α, aHIF may not explain the hypoxia-mediated inhibition of HIF-2α observed in our study. We are currently investigating the role of aHIF in hypoxic and cytokine-stimulated RA FLS.

Our study thus confirms previous studies reporting that HIF-1 represents a convergence point for inflammatory and hypoxic signalling in RA FLS, since we found that both Th1 cytokines and hypoxia induced HIF-1 protein as previously reported and this effect was additive when cells were co-stimulated [21]. We extended existing studies by showing additive effects of Th1 cytokines and hypoxia at the HIF-1 DNA binding activity level. Surprisingly, this cytokine-mediated increase in HIF-1 activity did not necessarily lead to induction of the four chosen downstream HIF-1 target genes in either normoxic or hypoxic RA FLS. In agreement with previous work [18,22], we found that VEGF was induced in a HIF-1-dependent manner by both of the Th1 cytokines tested in an additive (TNFα) and synergistic (IL-1β) fashion with hypoxia, as would be expected if both stimuli converge on HIF-1 with subsequent downstream target gene induction. Similarly, EFNA3 was induced by Th1 cytokines, albeit modestly, in a HIF-1-dependent manner, with IL-1β exerting an additive effect on EFNA3 expression in hypoxia. In contrast however, when TNFα were added to supernatants of RA FLS cultured under normoxic or hypoxic conditions, a strong negative effect on RA FLS-mediated expression of ANGPTL-4 was observed, despite the ability of Th1 cytokines to induce large amounts of active HIF-1 protein. Expression of the leptin gene, which was predominantly regulated by HIF-2, was also inhibited by TNFα in hypoxic RA FLS. These negative effects on adipokine expression were not HIF mediated, as we demonstrated with siRNA oligonucleotides against HIF isoforms. A complex picture therefore emerges from our study, in which angiogenic HIF target gene expression does not necessarily correlate positively with the level of HIF activity as is the case for VEGF in RA FLS.

The suppressive effects of combined hypoxia and TNFα on angiogenesis were further reflected in our findings that supernatants from RA FLS subjected to both stimuli did not induce tubule formation by HMEC-1 cells in a matrigel assay above controls. This observation could be due to the downregulation of potent mediators of synovial angiogenesis; for instance, adipokines. In contrast, supernatants from RA FLS stimulated with either TNFα or hypoxia as single stimuli induced angiogenesis above that of unstimulated cells, with hypoxia having the greatest effect on angiogenesis. These data suggest that the development of tubules is induced in areas of the RA synovial tissue where either inflammation or hypoxia dominates but is suppressed where inflammation and hypoxia co-exist, in spite of significant amounts of TNFα-induced HIF-1 and VEGF.

Our study confirmed that IL-4 stimulated RA FLS express VEGF in concordance with previous literature [46], but also highlighted a novel finding that IL-4 strongly induced ANGPTL-4 expression in a HIF-independent manner under both normoxia and hypoxia. In contrast, we found that IL-4 could completely abrogate hypoxia-induced leptin expression by RA FLS. In agreement with a study where IL-4 was shown to be proangiogenic in murine lungs *in vivo *under hypoxic conditions [47], we found supernatants from RA FLS co-stimulated with IL-4 and hypoxia to have even stronger functional angiogenic activity than supernatants from cells stimulated with IL-4 alone. These supernatants contained much less VEGF protein than supernatants from TNFα and hypoxia-stimulated cells.

In contrast to VEGF, the levels of ANGPTL-4 present in the RA FLS supernatants correlated with the degree of tubule formation by HMEC-1. ANGPTL-4 has previously been shown to stimulate tubule formation in a human umbilical vein endothelial cell-based matrigel assay in the absence of additional growth factors [43] and to induce anti-apoptotic activity in human vascular endothelial cells [48]. Moreover, ANGPTL-4 can induce a proangiogenic response in the chicken chorio-allantoic membrane, an effect that was shown not to require the presence of VEGF [49]. The presence of high levels of ANGPTL-4 might therefore contribute to the proangiogenic effects observed in the supernatants from IL-4-stimulated RA FLS. Similarly, the observed reduction in angiogenesis with hypoxia and TNFα-treated cell supernatants, when compared with effects of hypoxic supernatants, may be a consequence of adipokine downregulation by TNFα.

This is not the first time that negative regulation of angiogenic mediators by Th1 cytokines has been described in RA FLS. Recent work demonstrates a negative effect of IL-1β on matrix metalloproteinase-13 expression in hypoxic RA FLS [50]. In contrast to the HIF-independent downregulation of adipokines that we observed in hypoxic RA FLS, the work by Lee and colleagues demonstrated the involvement of HIF-1 in hypoxia-mediated downregulation of matrix metalloproteinase-13. Our data suggest that other overriding signalling pathways are induced in RA FLS, which can circumvent the strong induction of HIF-1 following stimulation with combined Th1 cytokines and hypoxia. This could possibly involve PPAR-α and PPAR-γ, established regulators of ANGPTL-4 in adipose tissue [51,52]. In addition to peroxisome proliferator-activated receptor response elements in the promoter region of ANGPTL-4, transforming growth factor β has been shown to regulate ANGPTL-4 via an enhancer element located ∼8 kb upstream of the transcriptional start site involving SMAD3, ETS1, RUNX, and AP-1 transcription factors [53,54]. We are presently investigating the signalling routes by which TNFα exerts its negative effects on adipokines in RA FLS.

The ability of TNFα to inhibit ANGPTL-4 expression was specific for RA FLS because normal HSF stimulated with the same concentration of TNFα exhibited a strong induction of ANGPTL-4. In contrast, the ability by IL-4 to upregulate ANGPTL-4 was shared by both RA FLS and HSF. These observations suggest that the inhibitory effect of TNFα is mediated via a decline in ANGPTL-4 gene expression, and moreover that it may be a disease-specific effect. Decreased expression of ANGPTL-4 might contribute to the transformed phenotype that characterises RA FLS [4,25]. Although ANGPTL-4 was identified in a gene expression profiling analysis as one of the most highly expressed genes in early CIA (day 28), this expression subsided with time (day 49) [43] - perhaps suggesting that RA FLS lose the ability to express ANGPTL-4 as disease progresses, with possible impact on blood vessel formation. The RA FLS we used are from patients with established RA of long duration.

The process of blood vessel maturation from immature vessels involves recruitment of perivascular cells, assembly of the basement membrane and establishment of tight and adherens junctions. Failure to form functional mature vessels is known to contribute to oedema formation, swelling and inflammation in RA joints (reviewed in [55]). Interestingly, recent work in *angptl-4*-deficient mice has shown that ANGPTL-4 is important in vessel maturation because mice lacking ANGPTL-4 exhibited disruption of endothelial adherens junctions and pericyte coverage, with impaired angiogenesis and vascular leakage as a result [56,57]. RA synovium contains a significant fraction of neoangiogenic, immature and leaky blood vessels that may be observed from the early stages of RA [6]. The presence or density of immature vessels is significantly increased in patients with longer disease duration, higher activity and severity, and stronger inflammatory cell infiltration [6]. Interestingly, immature vessels are depleted in response to anti-TNF therapy, highlighting the co-dependency of angiogenesis and inflammation [6]. Based on our findings we speculate that in areas of the synovium where hypoxia and Th1-driven inflammation co-exist there would be an excessive, pathological HIF-1-mediated response with elevated VEGF production, yet suppression of functional angiogenesis with immature vessel formation, a well-known effect of elevated VEGF [58]. If there is suppression of expression of angiogenic factors necessary for vessel maturation such as ANGPTL-4, the final outcome may thus be leaky and immature vessels. This is supported by a study showing that ANGPTL-4 decreases VEGF-induced vascular leakage in the Miles assay, which measures extravasation of Evans Blue dye from vessels in mouse back skin [58]. In contrast, Th2 cytokine therapy might ameliorate inflammation in CIA in hypoxic areas of the synovium, through the formation of mature vessels by overriding the local anti-angiogenic effects of hypoxia combined with Th1 cytokines and by inducing factors such as ANGPTL-4 resulting in vessel maturation. Elucidating the difference in the angiogenic gene profile induced by Th1 and Th2 cytokines in hypoxia is thus of great importance, because such differences may account for the pathological ratio of mature to immature vessels in RA depending on the type of angiogenic factors they induce or inhibit. We are currently investigating the significance of TNFα-induced inhibition of adipokines in RA FLS-mediated angiogenesis, with specific interest in defining the function of ANGPTL-4.

## Conclusion

In the present study we have demonstrated that Th1 cytokines in combination with hypoxia are not sufficient to induce angiogenic activity by RA FLS despite inducing HIF-1 activation and VEGF production. In contrast, Th2 cytokines induce proangiogenic activity in normoxia and hypoxia, despite their inability to activate HIFs, highlighting the complex relationships between hypoxia, angiogenesis and inflammation in RA. Furthermore, we have unmasked novel inhibitory effects of TNFα in combination with hypoxia on altering gene expression and on the functional angiogenic properties of RA FLS that have not been described elsewhere. These negative effects may influence the degree of pathological vessel formation in the RA synovium and hence the clinical response of RA patients on anti-TNFα treatment. Finally, our results highlight the potential role of ANGPTL-4 in regulating RA synovial angiogenesis through its differential regulation by proinflammatory and anti-inflammatory cytokines.

## Abbreviations

ANGPTL: angiopoietin-like; CIA: collagen-induced arthritis; DMEM: Dulbecco's modified Eagle's medium; DMOG: dimethyloxalylglycine; DMSO: dimethyl sulfoxide; EFNA3: ephrin A3; ELISA: enzyme-linked immunosorbent assay; FLS: fibroblast-like synoviocytes; HIF: hypoxia-inducible factor; HMEC: human microvascular endothelial cell; HRE: hypoxia response element; HSF: human skin fibroblast; IL: interleukin; IFN: interferon; mAb: monoclonal antibody; ; PBS: phosphate-buffered saline; PCR: polymerase chain reaction; RA: rheumatoid arthritis; siRNA: small interfering RNA; Th: T-helper cell; TNF: tumour necrosis factor; UTR: untranslated region; VEGF: vascular endothelial growth factor.

## Competing interests

The authors declare that they have no competing interests.

## Authors' contributions

HL, EMP and MF designed the study. HL and EMP wrote the manuscript. BM contributed to the siRNA knockdown studies. TLK assisted with generating the ELISA data. All authors read and approved the final manuscript.

## Supplementary Material

Additional file 1 **Table presenting the genes on Human Angiogenesis RT² Profiler™ PCR Arrays upregulated or downregulated by hypoxia in RA FLS from a single patient**. PCR array results presented as the fold-change in gene expression versus normoxia. Click here for file

Additional file 2 **Figures presenting supplementary data**. Click here for file

## References

[B1] Feldmann M Brennan FM Foxwell BM Maini RN The role of TNF alpha and IL-1 in rheumatoid arthritis Curr Dir Autoimmun 2001 3 188 199 11791466 10.1159/000060522

[B2] Sivakumar B Akhavani MA Winlove CP Taylor PC Paleolog EM Kang N Synovial hypoxia as a cause of tendon rupture in rheumatoid arthritis J Hand Surg Am 2008 33 49 58 10.1016/j.jhsa.2007.09.002 18261665

[B3] FitzGerald O Soden M Yanni G Robinson R Bresnihan B Morphometric analysis of blood vessels in synovial membranes obtained from clinically affected and unaffected knee joints of patients with rheumatoid arthritis Ann Rheum Dis 1991 50 792 796 10.1136/ard.50.11.792 1772295 PMC1004560

[B4] Mor A Abramson SB Pillinger MH The fibroblast-like synovial cell in rheumatoid arthritis: a key player in inflammation and joint destruction Clin Immunol 2005 115 118 128 10.1016/j.clim.2004.12.009 15885632

[B5] Koch AE Angiogenesis as a target in rheumatoid arthritis Ann Rheum Dis 2003 62 Suppl 2 ii60 ii67 14532152 10.1136/ard.62.suppl_2.ii60 PMC1766740

[B6] Izquierdo E Canete JD Celis R Santiago B Usategui A Sanmarti R Del Rey MJ Pablos JL Immature blood vessels in rheumatoid synovium are selectively depleted in response to anti-TNF therapy PLoS One 2009 4 e8131 10.1371/journal.pone.0008131 19956574 PMC2779850

[B7] Szekanecz Z Koch AE Mechanisms of disease: angiogenesis in inflammatory diseases Nat Clin Pract Rheumatol 2007 3 635 643 10.1038/ncprheum0647 17968334

[B8] Cha HS Bae EK Koh JH Chai JY Jeon CH Ahn KS Kim J Koh EM Tumor necrosis factor-alpha induces vascular endothelial growth factor-C expression in rheumatoid synoviocytes J Rheumatol 2007 34 16 19 17216674

[B9] Jackson JR Minton JA Ho ML Wei N Winkler JD Expression of vascular endothelial growth factor in synovial fibroblasts is induced by hypoxia and interleukin 1β J Rheumatol 1997 24 1253 1259 9228120

[B10] Maini RN Taylor PC Paleolog E Charles P Ballara S Brennan FM Feldmann M Anti-tumour necrosis factor specific antibody (infliximab) treatment provides insights into the pathophysiology of rheumatoid arthritis Ann Rheum Dis 1999 58 Suppl 1 I56 I60 10577974 10.1136/ard.58.2008.i56 PMC1766574

[B11] Yin G Liu W An P Li P Ding I Planelles V Schwarz EM Min W Endostatin gene transfer inhibits joint angiogenesis and pannus formation in inflammatory arthritis Mol Ther 2002 5 (Pt 1) 547 554 11991745 10.1006/mthe.2002.0590

[B12] Semenza GL Wang GL A nuclear factor induced by hypoxia via de novo protein synthesis binds to the human erythropoietin gene enhancer at a site required for transcriptional activation Mol Cell Biol 1992 12 5447 5454 1448077 10.1128/mcb.12.12.5447-5454.1992 PMC360482

[B13] Giatromanolaki A Sivridis E Maltezos E Athanassou N Papazoglou D Gatter KC Harris AL Koukourakis MI Upregulated hypoxia inducible factor-1α and -2α pathway in rheumatoid arthritis and osteoarthritis Arthritis Res Ther 2003 5 R193 R201 10.1186/ar756 12823854 PMC165055

[B14] Brouwer E Gouw AS Posthumus MD van Leeuwen MA Boerboom AL Bijzet J Bos R Limburg PC Kallenberg CG Westra J Hypoxia inducible factor-1-alpha (HIF-1α) is related to both angiogenesis and inflammation in rheumatoid arthritis Clin Exp Rheumatol 2009 27 945 951 20149310

[B15] Takeda N O'Dea EL Doedens A Kim JW Weidemann A Stockmann C Asagiri M Simon MC Hoffmann A Johnson RS Differential activation and antagonistic function of HIF-α isoforms in macrophages are essential for NO homeostasis Genes Dev 2010 24 491 501 10.1101/gad.1881410 20194441 PMC2827844

[B16] Westra J Brouwer E Bos R Posthumus MD Doornbos-van der Meer B Kallenberg CG Limburg PC Regulation of cytokine-induced HIF-1α expression in rheumatoid synovial fibroblasts Ann N Y Acad Sci 2007 1108 340 348 10.1196/annals.1422.035 17893997

[B17] Albina JE Mastrofrancesco B Vessella JA Louis CA Henry WL Jr Reichner JS HIF-1 expression in healing wounds: HIF-1α induction in primary inflammatory cells by TNF-α Am J Physiol Cell Physiol 2001 281 C1971 C1977 11698256 10.1152/ajpcell.2001.281.6.C1971

[B18] Hellwig-Burgel T Rutkowski K Metzen E Fandrey J Jelkmann W Interleukin-1β and tumor necrosis factor-alpha stimulate DNA binding of hypoxia-inducible factor-1 Blood 1999 94 1561 1567 10477681

[B19] Coimbra IB Jimenez SA Hawkins DF Piera-Velazquez S Stokes DG Hypoxia inducible factor-1 alpha expression in human normal and osteoarthritic chondrocytes Osteoarthritis Cartilage 2004 12 336 345 10.1016/j.joca.2003.12.005 15023385

[B20] He Y Fan J Lin H Yang X Ye Y Liang L Zhan Z Dong X Sun L Xu H The anti-malaria agent artesunate inhibits expression of vascular endothelial growth factor and hypoxia-inducible factor-1α in human rheumatoid arthritis fibroblast-like synoviocyte Rheumatol Int 2011 31 53 60 10.1007/s00296-009-1218-7 19859713

[B21] Westra J Brouwer E Bouwman E Doornbos-van der Meer B Posthumus MD van Leeuwen MA Limburg PC Ueda Y Kallenberg CG Role for CaMKII inhibition in rheumatoid arthritis: effects on HIF-1-induced VEGF production by rheumatoid synovial fibroblasts Ann N Y Acad Sci 2009 1173 706 711 10.1111/j.1749-6632.2009.04736.x 19758219

[B22] Berse B Hunt JA Diegel RJ Morganelli P Yeo K Brown F Fava RA Hypoxia augments cytokine (transforming growth factor-beta (TGF-β) and IL-1)-induced vascular endothelial growth factor secretion by human synovial fibroblasts Clin Exp Immunol 1999 115 176 182 10.1046/j.1365-2249.1999.00775.x 9933439 PMC1905193

[B23] Woods JM Tokuhira M Berry JC Katschke KJ Jr Kurata H Damergis JA Jr Arai K Koch AE Interleukin-4 adenoviral gene therapy reduces production of inflammatory cytokines and prostaglandin E_2 _by rheumatoid arthritis synovium ex vivo J Investig Med 1999 47 285 292 10431483

[B24] Horsfall AC Butler DM Marinova L Warden PJ Williams RO Maini RN Feldmann M Suppression of collagen-induced arthritis by continuous administration of IL-4 J Immunol 1997 159 5687 5696 9548513

[B25] Pap T Muller-Ladner U Gay RE Gay S Fibroblast biology. Role of synovial fibroblasts in the pathogenesis of rheumatoid arthritis Arthritis Res 2000 2 361 367 10.1186/ar113 11094449 PMC130137

[B26] Firestein GS Invasive fibroblast-like synoviocytes in rheumatoid arthritis. Passive responders or transformed aggressors? Arthritis Rheum 1996 39 1781 1790 10.1002/art.1780391103 8912499

[B27] Arnett FC Edworthy SM Bloch DA McShane DJ Fries JF Cooper NS Healey LA Kaplan SR Liang MH Luthra HS The American Rheumatism Association 1987 revised criteria for the classification of rheumatoid arthritis Arthritis Rheum 1988 31 315 324 10.1002/art.1780310302 3358796

[B28] Brennan FM Chantry D Jackson AM Maini RN Feldmann M Cytokine production in culture by cells isolated from the synovial membrane J Autoimmun 1989 2 Suppl 177 186 2505790 10.1016/0896-8411(89)90129-7

[B29] Uchida T Rossignol F Matthay MA Mounier R Couette S Clottes E Clerici C Prolonged hypoxia differentially regulates hypoxia-inducible factor (HIF)-1α and HIF-2α expression in lung epithelial cells: implication of natural antisense HIF-1α J Biol Chem 2004 279 14871 14878 10.1074/jbc.M400461200 14744852

[B30] Livak KJ Schmittgen TD Analysis of relative gene expression data using real-time quantitative PCR and the 2(-ΔΔC(T)) method Methods 2001 25 402 408 10.1006/meth.2001.1262 11846609

[B31] Del Rey MJ Izquierdo E Usategui A Gonzalo E Blanco FJ Acquadro F Pablos JL The transcriptional response of normal and rheumatoid arthritis synovial fibroblasts to hypoxia Arthritis Rheum 2010 62 3584 3594 10.1002/art.27750 20848564

[B32] Ambrosini G Nath AK Sierra-Honigmann MR Flores-Riveros J Transcriptional activation of the human leptin gene in response to hypoxia. Involvement of hypoxia-inducible factor 1 J Biol Chem 2002 277 34601 34609 10.1074/jbc.M205172200 12084725

[B33] Cascio S Bartella V Auriemma A Johannes GJ Russo A Giordano A Surmacz E Mechanism of leptin expression in breast cancer cells: role of hypoxia-inducible factor-1α Oncogene 2008 27 540 547 10.1038/sj.onc.1210660 17653093

[B34] Grosfeld A Andre J Hauguel-De Mouzon S Berra E Pouyssegur J Guerre-Millo M Hypoxia-inducible factor 1 transactivates the human leptin gene promoter J Biol Chem 2002 277 42953 42957 10.1074/jbc.M206775200 12215445

[B35] Belanger AJ Lu H Date T Liu LX Vincent KA Akita GY Cheng SH Gregory RJ Jiang C Hypoxia up-regulates expression of peroxisome proliferator-activated receptor gamma angiopoietin-related gene (PGAR) in cardiomyocytes: role of hypoxia inducible factor 1α J Mol Cell Cardiol 2002 34 765 774 10.1006/jmcc.2002.2021 12099716

[B36] Fasanaro P D'Alessandra Y Di Stefano V Melchionna R Romani S Pompilio G Capogrossi MC Martelli F MicroRNA-210 modulates endothelial cell response to hypoxia and inhibits the receptor tyrosine kinase ligand Ephrin-A3 J Biol Chem 2008 283 15878 15883 10.1074/jbc.M800731200 18417479 PMC3259646

[B37] Merlos-Suarez A Batlle E Eph-ephrin signalling in adult tissues and cancer Curr Opin Cell Biol 2008 20 194 200 10.1016/j.ceb.2008.01.011 18353626

[B38] Brantley DM Cheng N Thompson EJ Lin Q Brekken RA Thorpe PE Muraoka RS Cerretti DP Pozzi A Jackson D Soluble Eph A receptors inhibit tumor angiogenesis and progression in vivo Oncogene 2002 21 7011 7026 10.1038/sj.onc.1205679 12370823

[B39] Bouloumie A Drexler HC Lafontan M Busse R Leptin, the product of Ob gene, promotes angiogenesis Circ Res 1998 83 1059 1066 10.1161/01.RES.83.10.1059 9815153

[B40] Lago F Dieguez C Gomez-Reino J Gualillo O Adipokines as emerging mediators of immune response and inflammation Nat Clin Pract Rheumatol 2007 3 716 724 10.1038/ncprheum0674 18037931

[B41] Gabriel SE Michaud K Epidemiological studies in incidence, prevalence, mortality, and comorbidity of the rheumatic diseases Arthritis Res Ther 2009 11 229 10.1186/ar2669 19519924 PMC2714099

[B42] Van Doornum S Brand C King B Sundararajan V Increased case fatality rates following a first acute cardiovascular event in patients with rheumatoid arthritis Arthritis Rheum 2006 54 2061 2068 10.1002/art.21932 16802340

[B43] Hermann LM Pinkerton M Jennings K Yang L Grom A Sowders D Kersten S Witte DP Hirsch R Thornton S Angiopoietin-like-4 is a potential angiogenic mediator in arthritis Clin Immunol 2005 115 93 101 10.1016/j.clim.2004.12.002 15870027

[B44] Muz B Larsen H Madden L Kiriakidis S Paleolog EM Prolyl hydroxylase domain enzyme-2 is the major player in regulating hypoxic responses in rheumatoid arthritis Arthritis Rheum 2012 64 2856 2867 10.1002/art.34479 22488178

[B45] Thrash-Bingham CA Tartof KD aHIF: a natural antisense transcript overexpressed in human renal cancer and during hypoxia J Natl Cancer Inst 1999 91 143 151 10.1093/jnci/91.2.143 9923855

[B46] Hong KH Cho ML Min SY Shin YJ Yoo SA Choi JJ Kim WU Song SW Cho CS Effect of interleukin-4 on vascular endothelial growth factor production in rheumatoid synovial fibroblasts Clin Exp Immunol 2007 147 573 579 10.1111/j.1365-2249.2006.03295.x 17302909 PMC1810499

[B47] Yamaji-Kegan K Su Q Angelini DJ Johns RA IL-4 is proangiogenic in the lung under hypoxic conditions J Immunol 2009 182 5469 5476 10.4049/jimmunol.0713347 19380795 PMC10204605

[B48] Kim I Kim HG Kim H Kim HH Park SK Uhm CS Lee ZH Koh GY Hepatic expression, synthesis and secretion of a novel fibrinogen/angiopoietin-related protein that prevents endothelial-cell apoptosis Biochem J 2000 346 Pt 3 603 610 10698685 PMC1220891

[B49] Le Jan S Amy C Cazes A Monnot C Lamande N Favier J Philippe J Sibony M Gasc JM Corvol P Angiopoietin-like 4 is a proangiogenic factor produced during ischemia and in conventional renal cell carcinoma Am J Pathol 2003 162 1521 1528 10.1016/S0002-9440(10)64285-X 12707035 PMC1851201

[B50] Lee YA Choi HM Lee SH Hong SJ Yang HI Yoo MC Kim KS Hypoxia differentially affects IL-1β-stimulated MMP-1 and MMP-13 expression of fibroblast-like synoviocytes in an HIF-1alpha-dependent manner Rheumatology (Oxford) 2012 51 443 450 10.1093/rheumatology/ker327 22123992

[B51] Kersten S Mandard S Tan NS Escher P Metzger D Chambon P Gonzalez FJ Desvergne B Wahli W Characterization of the fasting-induced adipose factor FIAF, a novel peroxisome proliferator-activated receptor target gene J Biol Chem 2000 275 28488 28493 10862772 10.1074/jbc.M004029200

[B52] Yoon JC Chickering TW Rosen ED Dussault B Qin Y Soukas A Friedman JM Holmes WE Spiegelman BM Peroxisome proliferator-activated receptor gamma target gene encoding a novel angiopoietin-related protein associated with adipose differentiation Mol Cell Biol 2000 20 5343 5349 10.1128/MCB.20.14.5343-5349.2000 10866690 PMC85983

[B53] Kaddatz K Adhikary T Finkernagel F Meissner W Muller-Brusselbach S Muller R Transcriptional profiling identifies functional interactions of TGF beta and PPAR beta/delta signaling: synergistic induction of ANGPTL4 transcription J Biol Chem 2010 285 29469 29479 10.1074/jbc.M110.142018 20595396 PMC2937979

[B54] Padua D Zhang XH Wang Q Nadal C Gerald WL Gomis RR Massague J TGFβ primes breast tumors for lung metastasis seeding through angiopoietin-like 4 Cell 2008 133 66 77 10.1016/j.cell.2008.01.046 18394990 PMC2390892

[B55] Middleton J Americh L Gayon R Julien D Aguilar L Amalric F Girard JP Endothelial cell phenotypes in the rheumatoid synovium: activated, angiogenic, apoptotic and leaky Arthritis Res Ther 2004 6 60 72 15059266 10.1186/ar1156 PMC400438

[B56] Galaup A Gomez E Souktani R Durand M Cazes A Monnot C Teillon J Le Jan S Bouleti C Briois G Protection against myocardial infarction and no-reflow through preservation of vascular integrity by angiopoietin-like 4 Circulation 2012 125 140 149 10.1161/CIRCULATIONAHA.111.049072 22086875

[B57] Perdiguero EG Galaup A Durand M Teillon J Philippe J Valenzuela DM Murphy AJ Yancopoulos GD Thurston G Germain S Alteration of developmental and pathological retinal angiogenesis in angptl4-deficient mice J Biol Chem 2011 286 36841 36851 10.1074/jbc.M111.220061 21832056 PMC3196087

[B58] Ito Y Oike Y Yasunaga K Hamada K Miyata K Matsumoto S Sugano S Tanihara H Masuho Y Suda T Inhibition of angiogenesis and vascular leakiness by angiopoietin-related protein 4 Cancer Res 2003 63 6651 6657 14583458

