# Supplementary material for: Differential effects of Th1 versus Th2 cytokines in combination with hypoxia on HIFs and angiogenesis in RA
Source: Arthritis Res Ther. 2012 Aug 6;14(4):R180. doi: 10.1186/ar3934 (PMC3580575; doi:10.1186/ar3934)
Supplement: Additional file 1 — Table presenting the genes on Human Angiogenesis RT² Profiler™ PCR Arrays upregulated or downregulated by hypoxia in RA FLS from a single patient. PCR array results presented as the fold-change in gene expression versus normoxia. [file ar3934-S1.PDF]

Additional file 1 **Genes on Human Angiogenesis RT² Profiler™ PCR Arrays up- or downregulated by hypoxia in RA FLS from a single patient**

GENE	4 hours		24 hours	
	Change	P	Change	P
V-akt murine thymoma viral oncogene homolog 1	-1.15	NS	-1.23	NS
Angiopoietin 1	-1.08	NS	1.71	NS
Angiopoietin 2	1.35	NS	-3.02	**
Angiopoietin-like 3	-1.16	NS	2.23	NS
Angiopoietin-like 4	18.96	*	9.79	**
Alanyl aminopeptidase	-1.25	NS	1.50	NS
Brain-specific angiogenesis inhibitor 1	1.14	NS	2.22	NS
CCL-11	1.08	NS	-8.51	**
CCL-2	-1.45	NS	-1.60	NS
Cadherin 5, VE-cadherin	-1.29	NS	-1.79	NS
Collagen, type XVIII, alpha 1	1.33	NS	-1.15	NS
Collagen, type IV, alpha 3	2.21	*	2.63	NS
CXCL-1	1.31	NS	-2.74	**
CXCL-10	1.46	NS	-26.61	***
CXCL-3	1.12	NS	-4.18	*
CXCL-5	1.59	NS	-1.98	NS
CXCL-6	1.20	NS	-2.81	***
CXCL-9	3.58	NS	-1.95	NS
ECGF-1	1.19	NS	1.01	NS
EDG-1	-1.11	NS	-1.11	NS

Ephrin-A1	1.02	NS	-1.44	NS
Ephrin-A3	2.96	*	2.47	*
Ephrin-B2	-1.14	NS	1.33	NS
Epidermal growth factor	-1.52	NS	-1.44	NS
Endoglin	1.09	NS	1.31	NS
EPH receptor B4	-1.26	NS	1.17	NS
Epiregulin	1.27	NS	1.93	NS
Fibroblast growth factor 1	1.04	NS	1.65	NS
Fibroblast growth factor 2	-1.26	NS	1.10	NS
Fibroblast growth factor receptor 3	1.34	NS	1.44	NS
C-fos induced growth factor (VEGF-D)	-1.26	NS	-1.96	NS
Fms-related tyrosine kinase 1	1.29	NS	2.91	NS
Heart and neural crest derivatives expressed 2	-1.55	NS	-1.51	NS
Hepatocyte growth factor	-1.04	NS	-1.18	NS
HIF-1α	-1.03	NS	-1.23	**
Heparanase	-1.06	NS	-2.00	**
ID-1	-1.09	NS	1.09	NS
ID-2	1.27	NS	2.63	NS
Interferon, alpha 1	-1.01	NS	3.02	NS
Interferon, beta 1, fibroblast	1.06	NS	2.44	NS
Interferon, gamma	1.29	NS	2.63	NS
Insulin-like growth factor 1	1.10	NS	-1.98	NS
Interleukin 1, beta	1.05	NS	-2.35	NS
Interleukin 6	2.00	*	-1.52	NS

Interleukin 8	2.17	*	-1.17	NS
Integrin, alpha V (CD51)	-1.34	NS	-1.29	NS
Integrin, beta 3 (CD61)	1.33	NS	1.52	NS
Jagged 1	1.15	NS	-1.26	NS
Kinase insert domain receptor	-1.09	NS	3.24	NS
Laminin, alpha 5	1.22	NS	2.66	NS
LECT-1	-1.09	NS	4.14	**
Leptin	62.25	*	140.68	***
Midkine	1.02	NS	-1.00	NS
Matrix metallopeptidase 2	1.01	NS	1.46	NS
Matrix metallopeptidase 9	1.29	NS	3.33	NS
Notch homolog 4	1.29	NS	2.63	NS
Neuropilin 1	-1.12	NS	1.21	NS
Neuropilin 2	1.02	NS	1.32	NS
Platelet-derived growth factor alpha polypeptide	-1.06	NS	1.03	NS
PECAM-1	-1.13	NS	4.66	NS
Platelet factor 4	1.56	NS	1.91	NS
Placental growth factor	1.92	NS	-2.18	NS
Plasminogen activator, urokinase	-1.30	NS	-1.25	NS
Plasminogen	2.59	NS	1.36	NS
Plexin domain containing 1	-1.09	NS	-1.03	NS
Prokineticin 2	1.29	NS	2.63	NS
Prostaglandin-endoperoxide synthase 1	-1.25	NS	-1.30	NS
Serpin peptidase inhibitor	1.53	NS	-1.56	NS

Sphingosine kinase 1	1.30	NS	1.65	NS
Stabilin 1	-1.43	NS	2.50	NS
TEK tyrosine kinase	-1.27	NS	-1.13	NS
TGFalpha	1.52	NS	2.63	NS
TGF β 1	-1.00	NS	3.08	NS
TGF β 2	1.16	NS	-2.91	**
TGF β receptor I (activin A)	-1.01	NS	1.64	NS
Thrombospondin 1	1.23	NS	-1.45	NS
Thrombospondin 2	2.06	*	-1.32	NS
TIMP-1	-1.03	NS	1.40	NS
TIMP-2	-1.09	NS	1.09	NS
TIMP-3	-1.87	NS	1.25	NS
TNF superfamily, member 2	-1.20	NS	-1.63	NS
Tumour necrosis factor, alpha-induced protein 2	1.40	NS	-1.27	NS
VEGF	3.41	**	11.28	*
VEGF-C	-1.04	NS	1.49	NS

The PCR array was used to screen cDNA from FLS obtained from a patient with RA incubated for either 4 or 24 hours in 21% (normoxia) or 1% oxygen (hypoxia). The ΔCt values were obtained using Sequence Detection Software version 1.9.1 and compared to a number of HKG: ActB (β-actin), 18S rRNA, HPRT1 (hypoxanthine phosphoribosyltransferase 1) and RPL13A (60S ribosomal protein L13a). Values are fold change *versus* normoxia. Genes which changed significantly ($p<0.05$) by a factor of ≥2 in response to hypoxia were analysed by paired Student's t-test comparing ΔCt values (normoxia *versus* hypoxia): * $p<0.05$, ** $p<0.001$, *** $p<0.001$.
