# Supplementary material for: Differential effects of Th1 versus Th2 cytokines in combination with hypoxia on HIFs and angiogenesis in RA
Source: Arthritis Res Ther. 2012 Aug 6;14(4):R180. doi: 10.1186/ar3934 (PMC3580575; doi:10.1186/ar3934)
Supplement: Additional file 2 — Figures presenting supplementary data. [file ar3934-S2.PDF]

Supplementary data

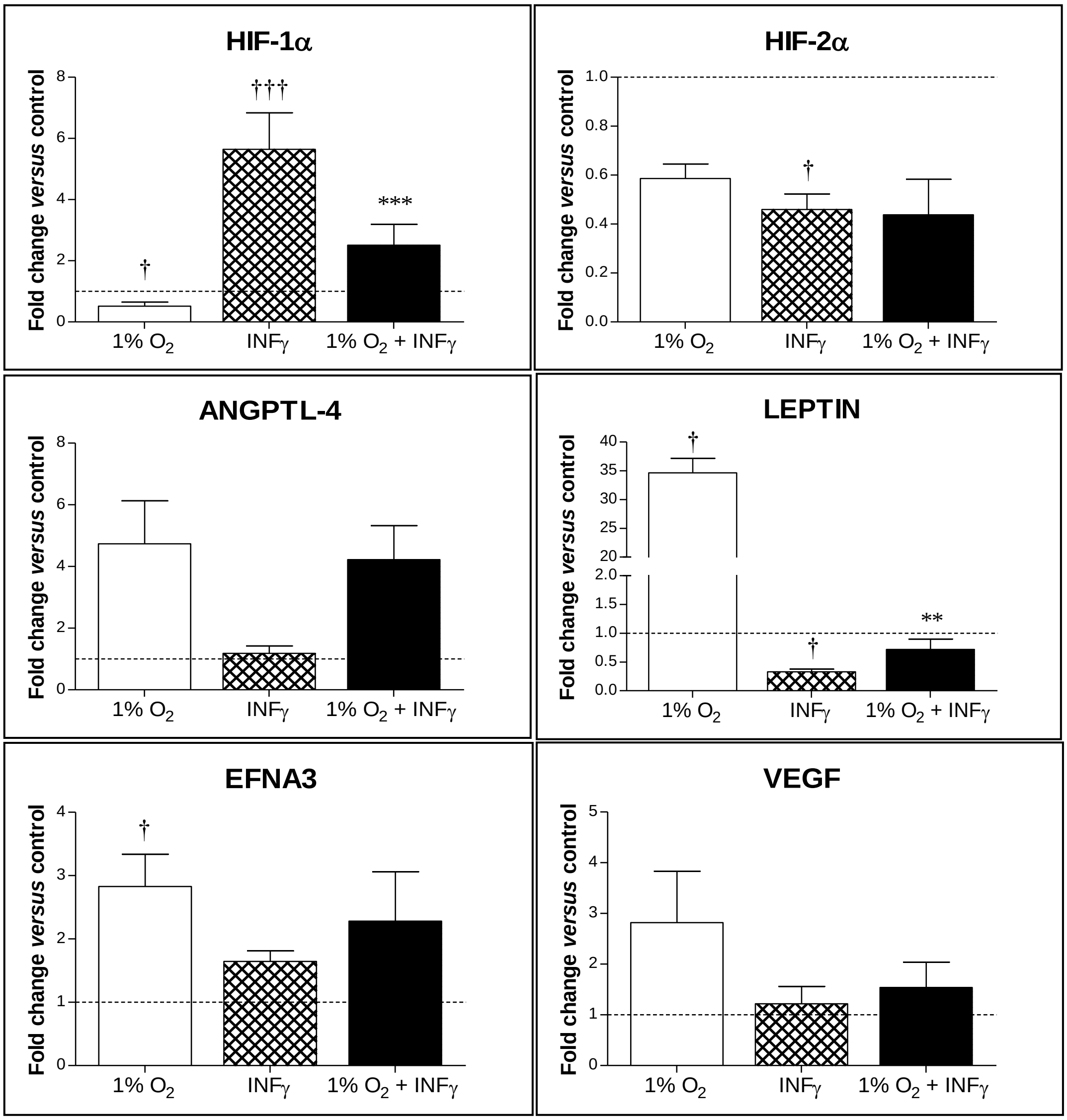

Figure 1 Q-PCR gene expression analysis of HIF-1α, -2α and angiogenic genes following RA FLS stimulation with INFγ and/or hypoxia

RA FLS were exposed for 24 hours to 1% O_2, 10ng/mL INFγ or a combination of both. Changes in mRNA of angiogenic genes in response to treatment are expressed as fold change relative to control unstimulated levels set as 1.0 (dotted line). Data are expressed as the mean SEM of ≥3 independent experiments with sample assayed in triplicate and were analysed using 1-way ANOVA with Bonferroni's post-hoc test for multiple comparisons *versus* control: † $p<0.05$, ††† $p<0.001$ or *versus* hypoxia alone: ** $p<0.01$, *** $p<0.001$.

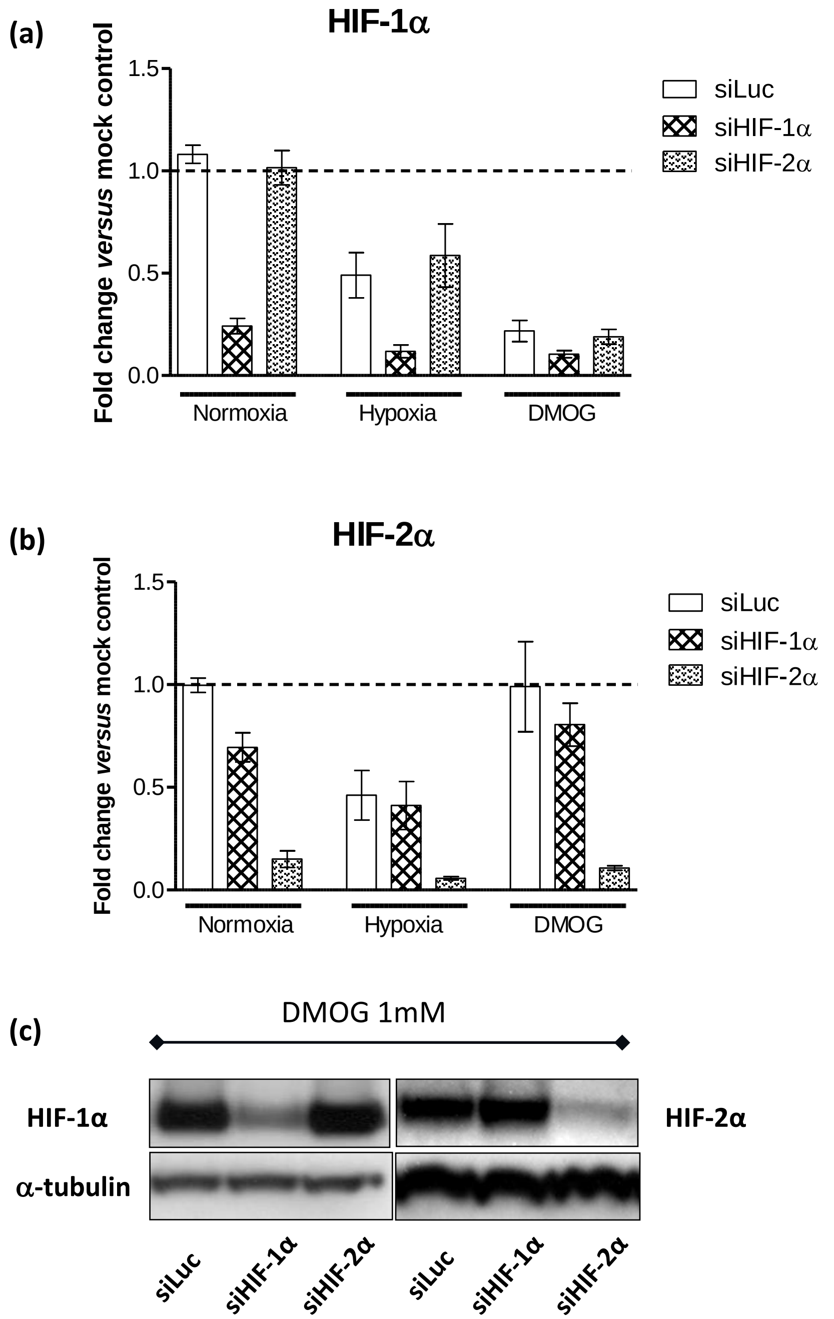

Figure 2 Q-PCR analysis and Western blotting of HIF-1 and HIF-2 following specific knock-down with siRNA oligonucleotides

RA FLS were transiently transfected with siRNA oligonucleotides complementary to HIF-1α (siHIF-1α) or HIF-2α (siHIF-2α) . An siRNA oligonucleotide complementary to siLuc was used as control. The cell cultures were subsequently exposed to either hypoxia (1% O_2) or 1mM DMOG. (a, b) Changes in mRNA in response to treatment are expressed as fold change relative to control unstimulated siLuc transfected normoxic cells set as 1.0 (dotted line). Data are expressed as the mean SEM of ≥3 independent experiments with sample assayed in triplicate. (c) Western blotting of samples from DMOG-stimulated cells, with α-tubulin shown as loading control.

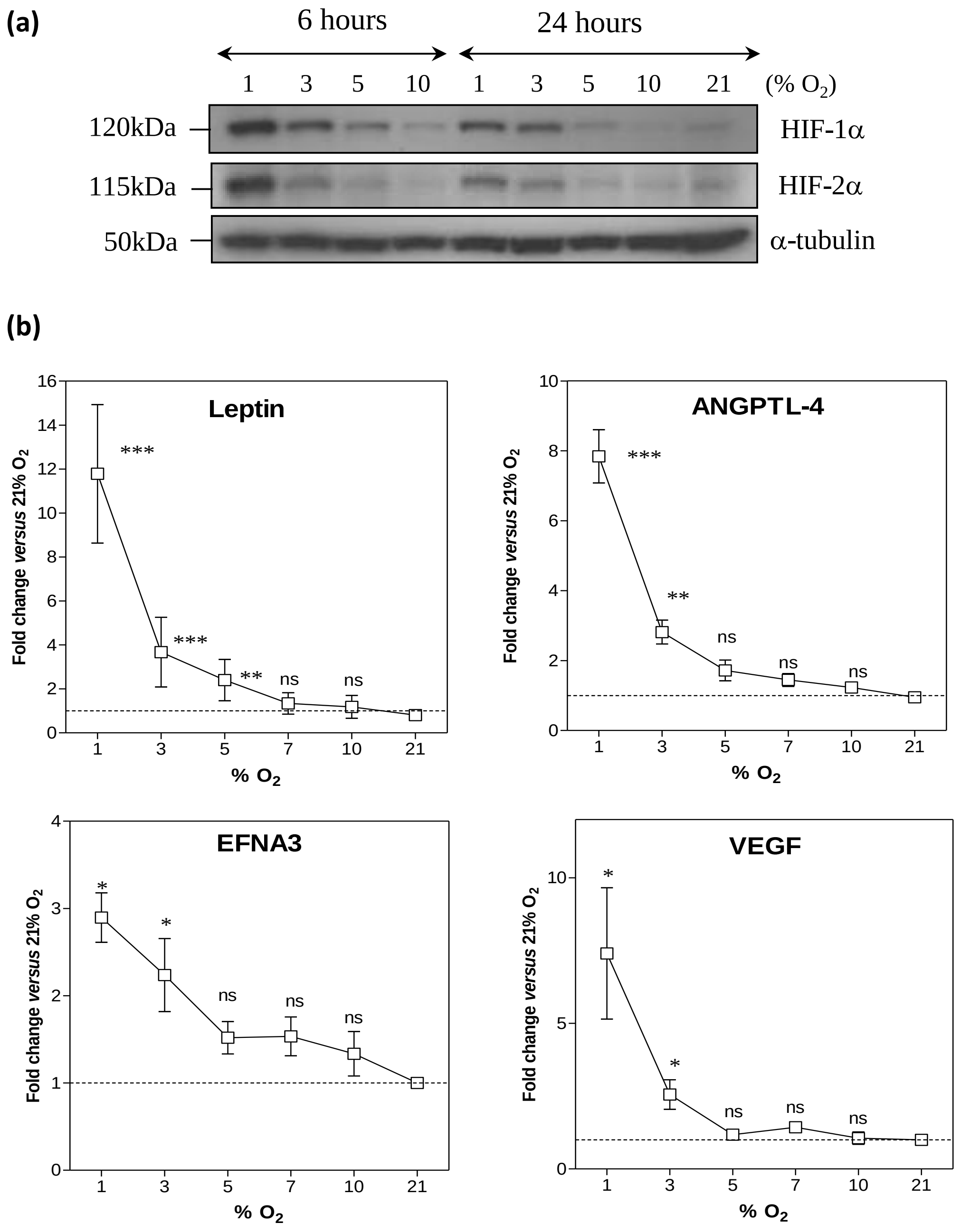

Figure 3 Changes in expression of HIF-1α, HIF-2α and HIF angiogenic target genes in human RA FLS at different oxygen tensions

RA FLS were exposed for 6 or 24 hours to different oxygen tensions, ranging from 1-21%O_2. (a) Western blotting showing changes in HIF-1α and HIF-2α protein with changing O_2 tensions with α-tubulin shown as loading control. (b) Changes in mRNA levels of angiogenic genes at various O_2 tensions expressed as fold change relative to levels in 21% O_2 set as 1.0 (dashed line). Data are expressed as the mean SEM of ≥3 independent experiments with sample assayed in triplicate, and were analysed by 1-way ANOVA *versus* normoxia: ns=not significant, * $p<0.05$, ** $p<0.01$, *** $p<0.001$.

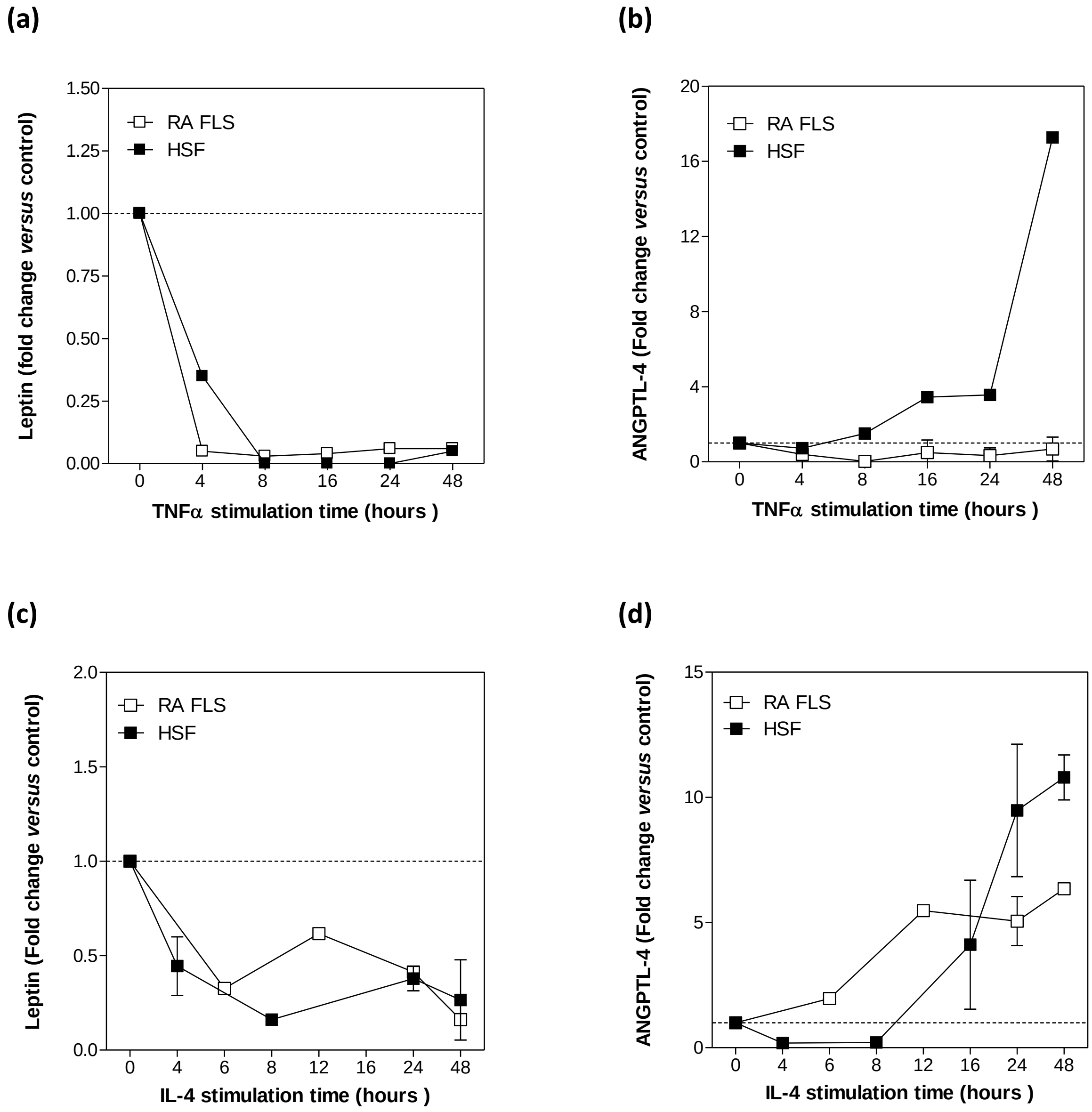

Figure 4 TNFα inhibits ANGPTL-4 expression in RA FLS but induces the adipokine in normal HSF

RA FLS or HSF were exposed to 10ng/mL of TNFα or IL-4 or left untreated for 0-48 hours. Total RNA was isolated and cDNA generated and the mRNA level of (a,c) leptin and (b,d) ANGPTL-4 was determined using Q-PCR. Changes in mRNA are expressed as fold change relative to levels in untreated samples set as 1.0 (dotted line).
